# Synthesis and Characterization of Amino-Functional Polyester Dendrimers Based On Bis-MPA with Enhanced Hydrolytic Stability and Inherent Antibacterial Properties

**DOI:** 10.1021/acs.biomac.2c01286

**Published:** 2023-01-23

**Authors:** Faridah Namata, Natalia Sanz del Olmo, Noemi Molina, Michael Malkoch

**Affiliations:** Department of Fibre and Polymer Technology, KTH Royal Institute of Technology, Teknikringen 56-68, 100 44 Stockholm, Sweden

## Abstract

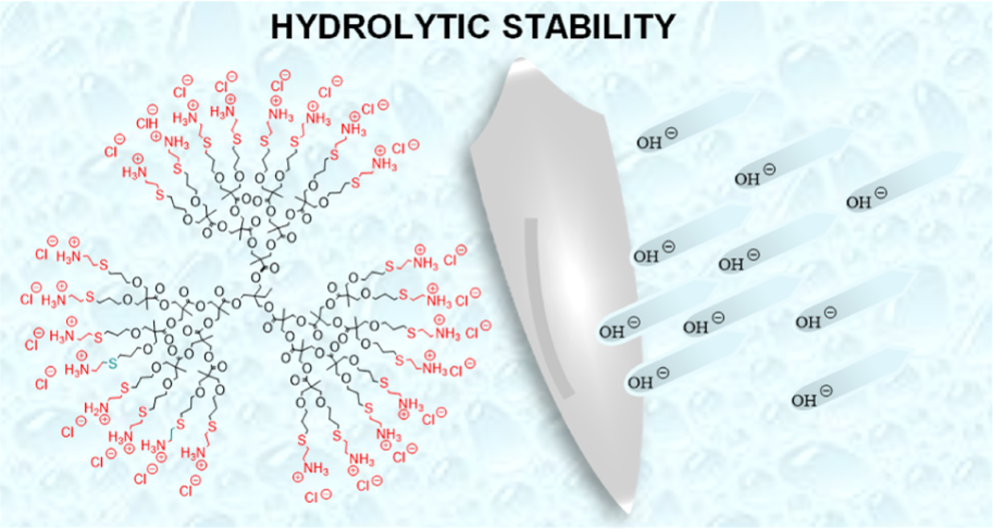

Polyester dendrimers based on 2,2 bis(hydroxymethyl)propionic acid have been reported to be degradable, non-toxic, and exhibit good antimicrobial activity when decorated with cationic charges. However, these systems exhibit rapid depolymerization, from the outer layer inwards in physiological neutral pHs, which potentially restricts their use in biomedical applications. In this study, we present a new generation of amine functional bis-MPA polyester dendrimers with increased hydrolytic stability as well as antibacterial activity for Gram-positive *Staphylococcus aureus* (*S. aureus*) and Gram-negative *Escherichia coli* (*E. coli*) and *Pseudomonas aeruginosa* (*P. aeruginosa*) planktonic bacteria strains. These new derivatives show generally good cytocompatibility for the concentrations they are active toward bacteria, in monocyte/macrophage-like cells (Raw 264.7), and human dermal fibroblasts. Fluoride - promoted esterification chemistry, anhydride chemistry, and click reactions were utilized to produce a library from generations 1–3 and with cationic peripheral groups ranging from 6 to 24 groups, respectively. The dendrimers were successfully purified using conventional purification techniques as well as characterized by matrix-assisted laser desorption ionization time-of-flight mass spectroscopy, nuclear magnetic resonance, and size exclusion chromatography. As proof of synthetic versatility, dendritic-linear-dendritic block copolymer were successfully synthesized to display cysteamine peripheral functionalities as well as the scaffolding ability with biomedically relevant lipoic acid and methoxy polyethylene glycol.

## Introduction

Dendrimers, as the main subgroup of dendritic polymers, are a class of synthetic polymers that stand out for being typically symmetrical, highly branched, and monodisperse. Due to their unique features, which include condensed branched structures with precise control of the size, shape, and multiple functional groups on their outer layer, there is unlimited potential for their use in biomedical applications.^1−4^ The synthesis of dendrimers is often based on a repeated sequence of robust organic reactions, where each successive reaction results in a higher dendritic generation that at least doubles in terms of both molecular weight and the number of peripheral groups. Tomalia and Newkome, independently, were one of the first authors to report the full synthesis and characterization of dendrimers named “true dendrimers”^5^ and cascade molecules,^6^ respectively. Thereafter, Tomalia was the first to commercialize one of the most commonly used dendrimers, poly(amidoamine) (PAMAM) dendrimers.

PAMAM dendrimers are one of the most researched families with respect to biomedical applications. They have been reported to be used as drug carriers,^7^ bioimaging contrasting agents in MRI,^8,9^ and gene delivery agents.^10^ It is imperative to assess biocompatibility and cytotoxicity of compounds when considering their potential for pharmaceutical and biomedical use. The cytotoxicity of PAMAM dendrimers in diverse mammalian cell lines have been studied.^11−14^ Byrne et al. reported that in a prolonged particle exposure time assay evaluation, amine functional PAMAM dendrimers demonstrated severe chronic toxicity effects as compared to the acute effects.^13^ They confirmed that the toxicity pathway leading to cell death was the accumulation of dendrimers in cell tissues.^14^ The reduced biocompatibility of amine functional PAMAM dendrimers can be attributed to their polycationic character^15^ and their accumulation in the cells or tissues due to their delayed degradation. Chemically, the slow degradation can be attributed to the amide interior of the dendrimer that is both hydrolytically stable under physiological pH as well as enzymatically stable in vivo. The latter represents a real challenge for researchers who strive to include structural modifications to create a new generation of self-immolative dendrimers that decompose upon a triggering event.^16^ In light of these limitations, there is interest in biodegradable dendrimers as a non-toxic alternative.

In this context, polyester dendrimers based on 2,2 bis(hydroxymethyl)propionic acid (bis-MPA) have arisen as promising alternatives as a consequence of some advantages, such as robust synthesis, structural versatility, good cytotoxicity profile, and degradation into non-toxic adducts.^1,2,17^ Fréchet and Szoka reported the synthesis and biological evaluation of various dendritic scaffolds based on bis-MPA, where they detailed toxicity evaluation and biodegradation under physiological conditions.^18,19^ Fadeel et al. carried out an extensive evaluation that related the biodegradation and cytotoxicity of bis-MPA dendrimers to their generation and their surface groups in comparison to hydroxyl and amine functional PAMAM dendrimers. This study revealed that the hydroxyl functional dendrimers were not toxic, while cationic PAMAM dendrimers were found to exert cytotoxicity. Additionally, the hydroxyl functional bis-MPA dendrimers were found to be more susceptible to degradation at pH 7.5 when compared with more acidic pH 4.5 conditions. Interestingly, the degradation was observed to occur through a mechanism of depolymerization, where the hydrolysis of ester bonds proceeds first from the peripheral layer inward toward the core.^20^ Pegylated bis-MPA dendrimer of second generation was also found to undergo similar degradation processes in which drastic fragmentation and full decomposition was observed after 15 days and 2 months, respectively.^21^

Moreover, highly charged cationic polymers have shown to be promising agents in killing bacteria and/or inhibiting their growth.^22^ Specifically, cationic dendrimers have been reported to show good antimicrobial activity against different bacteria strains.^23,24^ The mechanism suggested for cationic polymers killing bacteria involves interaction and sequential abruption of the negatively charged bacteria membrane.^25,26^ Amine functional bis-MPA dendrimers decorated with β-alanine were evaluated for their antimicrobial activity^27,28^ and degradability.^28^ The second generation β-alanine-functionalized bis-MPA dendrimer, with 12 positive charges, inhibited the growth of bacteria *Escherichia coli* while being nontoxic to cells at the same concentration.^28^ The hydrolytic evaluation showed rapid degradation through loss of β-alanine groups at physiological pH.^28^ Even though traditional bis-MPA dendrimers have a wide range of desired properties that fulfil the needs for application-driven research within the realm of biomedical applications, their hydrolytic depolymerization profile at pH 7 can be a limiting factor where stability is preferred for conjugation purposes or prolonged performance in a physiological environment.

In this work, we present a new family of amine-functionalized bis-MPA dendrimers that maintain the internal structural integrity at elevated pH. A divergent growth approach was employed, combining esterification for the generation build-up and thiol–ene click reactions, to synthesize polyester dendrimers of generation 1–3 (G1–G3). The final dendrimers displayed up to 24 primary amines at the outer layer. In addition to the exceptional hydrolytic stability of the dendrimers, antibacterial evaluation against Gram-negative and Gram-positive bacteria as well as cytotoxicity screening was conducted against human dermal fibroblast (hDF) and mouse monocyte cells (Raw 264.7). To prove the versatility of this strategy, amine-functionalized dendritic–linear–dendritic block copolymers (DLDs) were synthesized as an example of a different dendritic architecture. Additionally, to expand the application scope of the new family of cationic systems, biologically interesting compounds, such as lipoic acid and polyethylene glycol (PEG), were attached to the dendritic periphery. This was accomplished by the use of fluoride-promoted esterification (FPE) and anhydride chemistries.

## Experimental Section

### Materials

All materials and solvents were purchased from Sigma Aldrich and used as received unless otherwise noted. 2,2-Bis(methylol)propionic acid (bis-MPA) and trimethylolpropane (TMP) were kindly donated by Perstorp AB, Sweden. Methoxy PEG (mPEG_11_) acid was purchased from Polypure. The photoinitiator Irgacure 651, that is referred to as 2,2-dimethoxy-2-phenylacetophenone (DMPA), was purchased from Ciba Specialty Chemicals Inc. (Switzerland).

*Escherichia coli* 178 (*E. coli* 178) was kindly provided by Professor Paul Orndorff (North Carolina State University). *Staphylococcus aureus* 2569 (*S. aureus* 2569) and *Pseudomonas aeruginosa* 22644 (*P. aeruginosa* 22644) were purchased from DSMZ. hDF and mouse monocyte (Raw 264.7) cells were purchased from the American Tissue Culture Collection. For the tests of cell viability, Dulbecco’s modified Eagle medium (DMEM), fetal bovine serum (FBS), and the mixture of antibiotics penicillin/streptomycin were purchased from Thermo Fisher Scientific.

### Synthetic Protocols

All synthetic protocols are presented in the Supporting Information.

### Characterization Methods

#### Nuclear Magnetic Resonance

Analyses were performed using a Bruker AM nuclear magnetic resonance (NMR). ^1^H-NMR and ^13^C-NMR were recorded at 400 and 101 MHz, respectively. ^1^H-NMR spectra were acquired using a spectral window of 20 ppm, a relaxation delay of 1 s, and 16 scans with automatic lock and shimming. ^13^C-NMR spectra were acquired using a spectral window of 240 ppm, a relaxation delay of 2 s, and from 256 to 1024 scans. Analyses of the obtain spectra were conducted using MestReNova version 14.2.0-26256 (Mestrelab Research S.L 2020). All relevant NMR spectra can be found in the Supporting Information.

#### Matrix-Assisted Laser Desorption/Ionization

Analyses were performed using a Bruker UltrafleXtreme matrix-assisted laser desorption ionization time-of-flight (MALDI TOF)/TOF mass spectrometer (Bruker Daltonics, Bremen, Germany) equipped with a SmartbeamII laser (355 nm, UV) in the positive mode. Calibrations were performed using a peptide calibration standard (Bruker Daltonics, Bremen, Germany). Mass spectra were recorded with FlexControl and analyzed with FlexAnalysis Version 3.4 (Bruker Daltonics). 2,5-Dihydroxybenzoic acid was used as the matrix and prepared by dissolution at a concentration of 20 mg mL^–1^ in tetrahydrofuran. Analyte was dissolved at a concentration of 1 mg mL^–1^ in MeOH, for dendrimer characterization, and in phosphate-citrate buffers, for the dendrimer degradation. Samples were prepared at a ratio of 40:1 of the matrix and analyte, respectively. A 5 μL droplet was deposited on an MPT 284 Target ground steel TF Target plate purchased from Bruker Daltonics. All relevant MALDI spectra can be found in the Supporting Information.

#### Size Exclusion Chromatography

Analyses were performed in dimethylformamide with 0.01 M LiBr as the mobile phase at a flowrate of 0.2 mL min^–1^ at 35 °C. An ATOSOH EcoSEC HLC-8320GPC system was used, equipped with an EcoSEC RI detector and three columns (PSS PFG 5 μm; Microguard, 100, and 300 Å) (MW resolving range: 300–100,000 Da) from PSS GmbH. Sample solutions with a concentration in the range of 3–4 mg mL^–1^ were used. A conventional calibration method was created using narrow linear poly(ethylene glycol) or poly(methyl methacrylate) standards purchased from PSS range 800–202,000 Da. Corrections for flow rate fluctuations were made using toluene as an internal standard. PSS WinGPC Unity software version 7.2 was used to process data and graphs, where normalized and plotted in Origin 9.1.0 Sr1. All relevant size exclusion chromatography (SEC) spectra can be found in the Supporting Information.

### Degradation Evaluation

A 0.5 mM solution of G2-[Cys]_12_ bis-MPA dendrimer was prepared in phosphate-citrate buffers of pH 4.4, 5.4, 6.4, and 7.4 with an ionic strength of 0.1 M KCl and kept at 37 °C. The pH of the solutions was reconfirmed after the addition of the dendrimer. Aliquots were analyzed with MALDI by following the above-mentioned protocol at different times (0 h, 1 h, 3 h, 8 h, 1 day, 2 days, 7 days, 16 days, and 30 days). See Figure S9 in the Supporting Information.

### Minimum Inhibitory Concentration and Minimum Bactericidal Concentration

Minimum inhibitory concentration (MIC) and minimum bactericidal concentration (MBC) assays were used to evaluate the antibacterial activities of the cationic dendrimers toward *E. coli*, *S. aureus,* and *P. aeruginosa*. For MIC evaluation, samples were diluted with sterilized phosphate-buffered saline (PBS) using the double dilution method. Bacterial solutions at the log phase were diluted with MHB II broth to reach the concentration of 10^6^ CFU mL^–1^. After inoculation, equal volume (50 μL) of the microorganism was incubated with biocides and controls in sterile 96-well plates at 37 °C for 18 h with a shaking of 250 rpm. The final optical density was then measured using the Multiskan FC Microplate reader [Thermo Fisher scientific (Shanghai) instruments Co., Ltd.] using an OD of 620 nm to determine MIC. The negative controls comprise the inoculum, bacteria without biocides, and the culture medium, sample without an inoculum and biocide. Amoxicillin was used as a positive control. All concentrations and controls were evaluated in triplicate. Subsequently, to obtain the MBC, 5 μL of the MIC, 2× MIC, 4× MIC, and 8× MIC, as well as controls were deposited on a Petri dish containing a solid medium and being incubated for 24 h.

### Determination of Cytotoxicity

The cytotoxicity study was carried out toward Raw 264.7 and hDF cells maintained in DMEM containing 10 % FBS and 100 units mL^–1^ penicillin plus 100 mg mL^–1^ streptomycin under 5 % CO_2_ at 37 °C. For the cell viability evaluation, cells were washed with PBS 1× and harvested either with trypsin for hDF or by scraping for Raw 264.7. Afterward, cells were resuspended in complete DMEM, counted with a hemocytometer, and transferred 100 μL into 96-well plates at a concentration of 1 × 10^6^ cells mL^–1^. Cells were cultured for 24 h before treatment. The medium was replaced by a solution of dendrimers in the cell culture at concentrations 0.1–50 μM. Five parallel wells were set for each concentration. The cells were incubated for another 24 h following by the addition of Alamar Blue reagent. Fluorescence intensity was measured after 4 h using a plate reader (Tecan Infinite M200 Pro) at the wavelength of 560/590 nm (excitation/emission).

## Results and Discussion

The application of bis-MPA-based polyester dendrimers in the biomedical field is promising and founded on over 3 decades of application-driven research.^2,29^ However, their poor structural stability at physiological neutral pH can be considered as a limiting factor with respect to time-dependent performance. To elevate this platform to include desirable dendritic scaffolds that deliver consistent performance under physiological conditions, the inhibition of the known degradation process is of outmost importance, especially taking into consideration that the depolymerization process is initiated at the vulnerable ester bonds allocated at the exterior of traditional bis-MPA dendrimers. Consequently, we envisioned that the replacement of the exterior exposed bonds with more stable bonds could be a viable approach to manipulate their stability. As a result, we sought out a simple yet robust synthetic strategy toward stable bis-MPA dendrimers that capitalizes on maintaining the interior polyester structure, while introducing stable thioether bonds at the dendrimer corona displaying primary amine as suitable reactive groups for bioconjugation purposes.

### Physiological pH Stable Bis-MPA Dendrimers

**Scheme 1:**
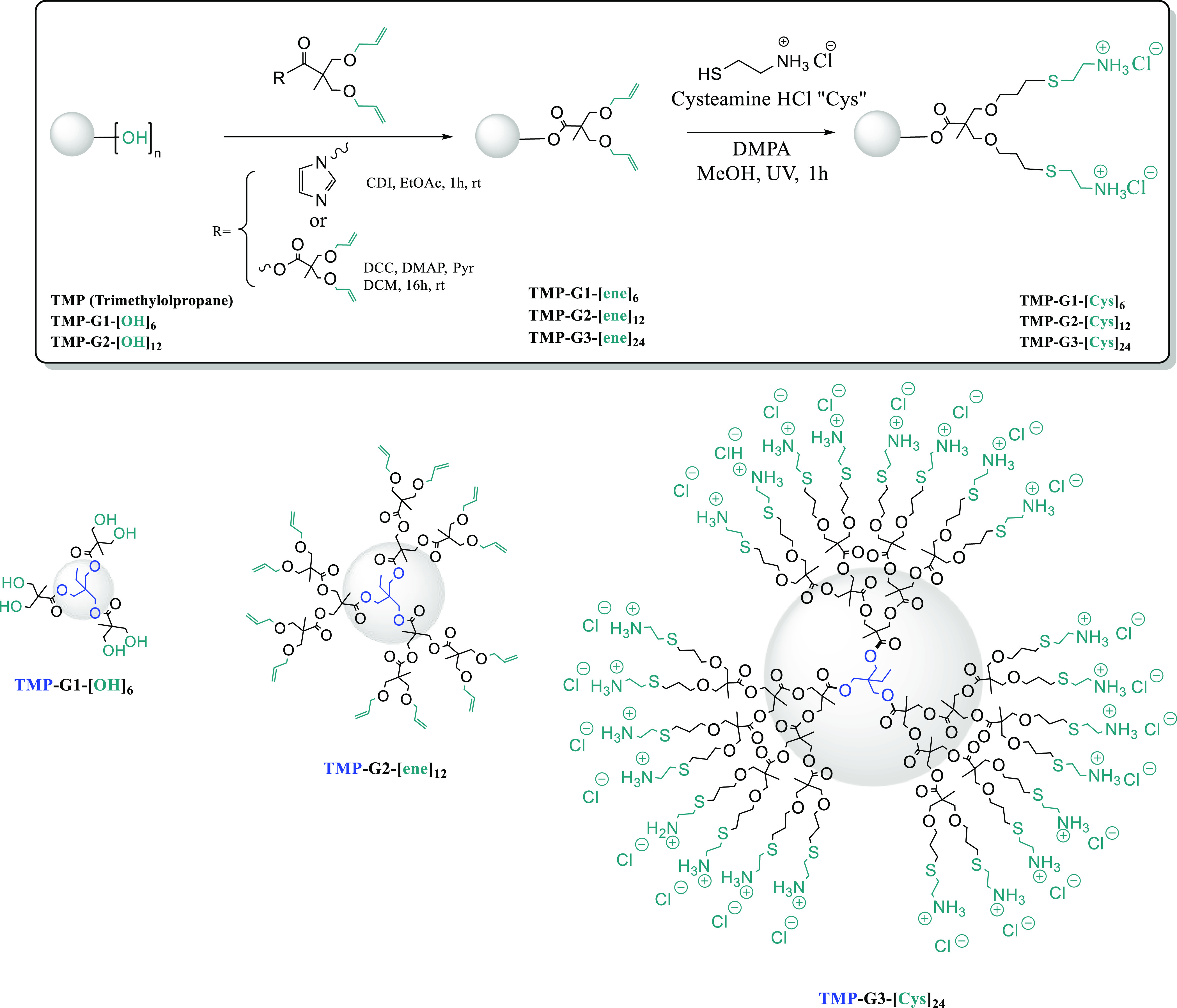
Synthetic Strategy for the Novel Family of Cationic Dendrimers Based on Bis-MPA

To accomplish this, traditional bis-MPA dendrimers were synthesized via a layer-by-layer divergent growth approach using an iterative combination of FPE and deprotection chemistry to obtain peripheral hydroxylated dendritic skeletons.^17,30^ Then after, the multiple representation of hydroxyls was esterified using bis-allylated bis-MPA (BAPA),^31^ for the introduction of allylic peripheral groups, as represented in Scheme 1. This was accomplished using FPE chemistry, in which imidazole-activated BAPA, was successful using *N*,*N*′-carbonyldiimidazole (CDI) for 1 h at room temperature and then in situ reacted with hydroxyls overnight in the presence of CsF as a soft inorganic base. After subsequent washes with NaHCO_3_ and NaHSO_4_, generation one (TMP-G1-[ene]_6_) and two (TMP-G2-[ene]_12_) were isolated as yellow oil in near quantitative yields. For higher generation dendrimers, complete functionalization via the FPE chemistry was found to require a larger molar ratio of CDI-activated BAPA. Consequently, the third-generation dendrimer (TMP-G3-[ene]_24_) was synthesized using anhydride-activated BAPA,^31^ in the presence of catalytic amount of 2-(dimethylamine) pyridine (DMAP) and excess of pyridine.

To showcase a new platform of functional polyester bis-MPA dendrimers that is stable at physiological pH, we sought out the introduction of protonated amines suitable for antimicrobial activity. These can be further neutralized to obtain strong nucleophiles for bioconjugation reactions. Therefore, peripheral allyls were reacted with cysteamine hydrochloride using thiol–ene click chemistry (TEC) in the presence of 2,2-dimethoxy-2-diphenylacetophenone (DMPA) as a radical initiator. The TEC reaction was highly efficient and full conversion of the allyls was accomplished within 1 h under exposure to UV light. Cysteamine-functionalized bis-MPA dendrimers of generation 1 to 3 (TMP-G1-[Cys]_6_, TMP-G2-[Cys]_12_, and TMP-G3-[Cys]_24_) displaying stable thioether bonds and with molecular weight up to 5381 Da and 24 reactive amines were successfully isolated. The purification included straightforward by-mass separation using Sephadex G-10, in which the dendrimers were obtained as sticky yellow solids in moderate yields.

**Figure 1:**
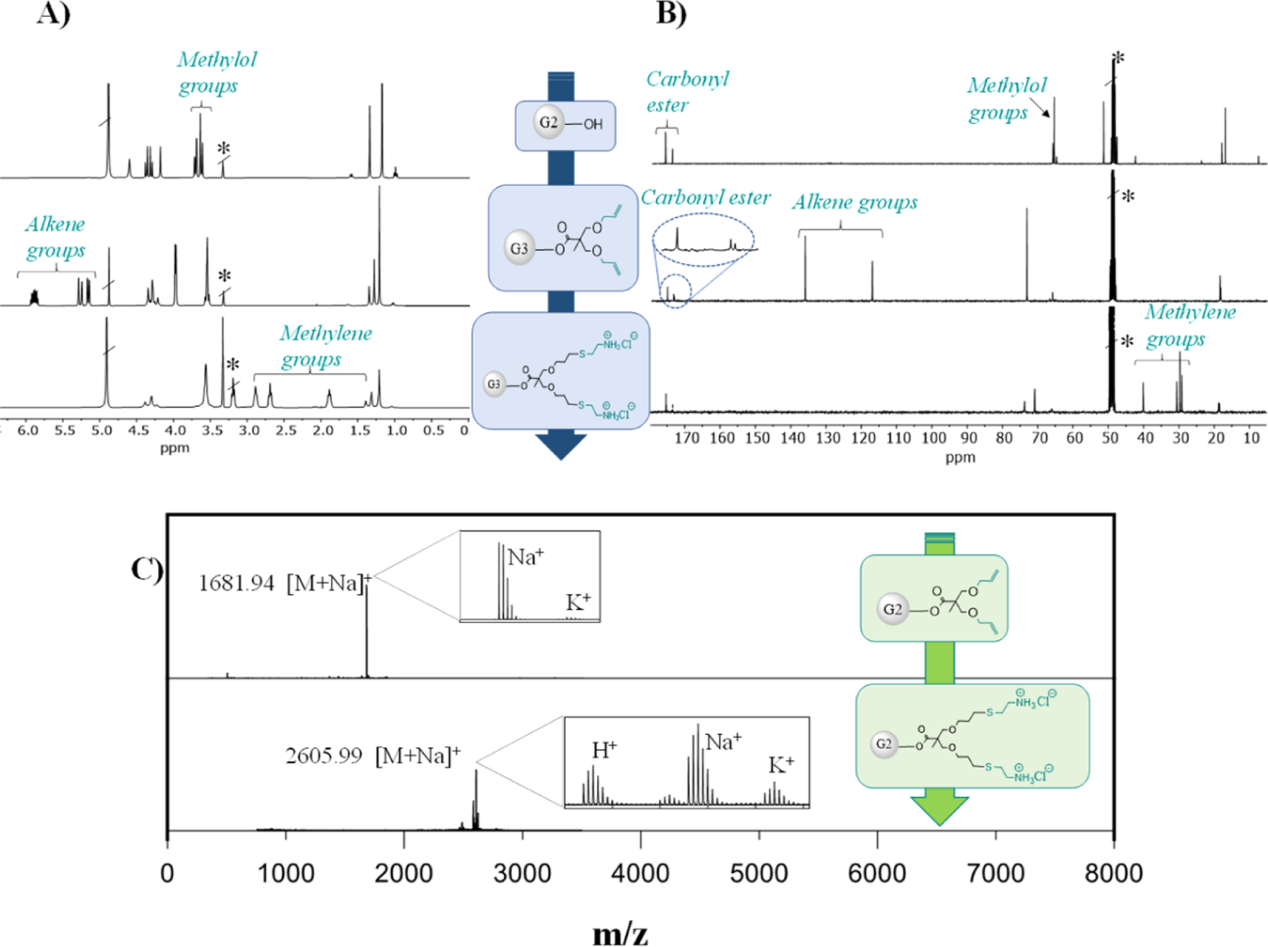
(A) ^1^H-NMR and (B) ^13^C-NMR spectra of the third-generation dendrimers (TMP-G3-[OH]_24_, TMP-G3-[ene]_24_, and TMP-G3-[Cys]_24_) in CD_3_OD (*). C) MALDI-TOF for the second-generation dendrimers (TMP-G2-[ene]_12_ and TMP-G2-[Cys]_12_ with theoretical values of [M + Na]_Theo_+: 1681.89 Da and [M + Na]_Theo_^+^: 2606.25 Da, respectively).

The monitoring of the sequence of reactions was conducted using a combination of NMR (Figure 1A,B) and MALDI (Figure 1C) techniques.

In the first step of esterification reaction, between the hydroxyl groups of the dendritic precursors and the carboxylic acid of the BAPA derivative, the appearance of new signals attributed to the protons of the alkene group at 5.89 and 5.22 ppm in ^1^H-NMR (Figure 1A) as well as at 136.2 and 117.2 ppm in ^13^C-NMR (Figure 1B) could be confirmed. At the same time, the resonances for the protons of the hydroxymethyl moieties are shifted during the esterification from 3.66 to 4.30 ppm. Additionally, the complete esterification of the dendrimer was also revealed by ^13^C-NMR with the presence of a new signal in the carbonyl region at around 173.4 ppm (Figure 1B). The progress of the TEC reaction was also monitored by NMR through the disappearance of the signals attributed to the protons of the alkene functionality. Moreover, after the reaction completion, new signals appeared in the range of 3.24–1.84 and 28.8–40.1 ppm for ^1^H-NMR (Figure 1A) and ^13^C-NMR (Figure 1B), respectively. These signals were attributed to the protons of the new methylene groups in the final cationic dendritic derivative. MALDI was further employed to corroborate on the structural integrity of the produced dendrimers as well as confirm the absence of incomplete functionalization during reaction progression (Figure 1C). The masses detected in MALDI were without the counterion negative hydrochloric acid but rather with either/and/or H^+^, Na^+^, and K^+^, as the instrument was set to the positive mode.

All intermediates with double bonds as functionalities have been fully characterized through NMR, MALDI, and SEC, confirming the structural perfection, monodispersity, and purity. For the final cationic derivatives, NMR and MALDI techniques have not been used; however, a good-quality SEC spectrum was not obtained as a consequence of poor solubility in the required solvents by the technique. More detailed synthetic protocols and characterization (^1^H-NMR, ^13^C-NMR, MALDI, and SEC) are described in the Supporting Information.

### Degradation Evaluation

**Figure 2:**
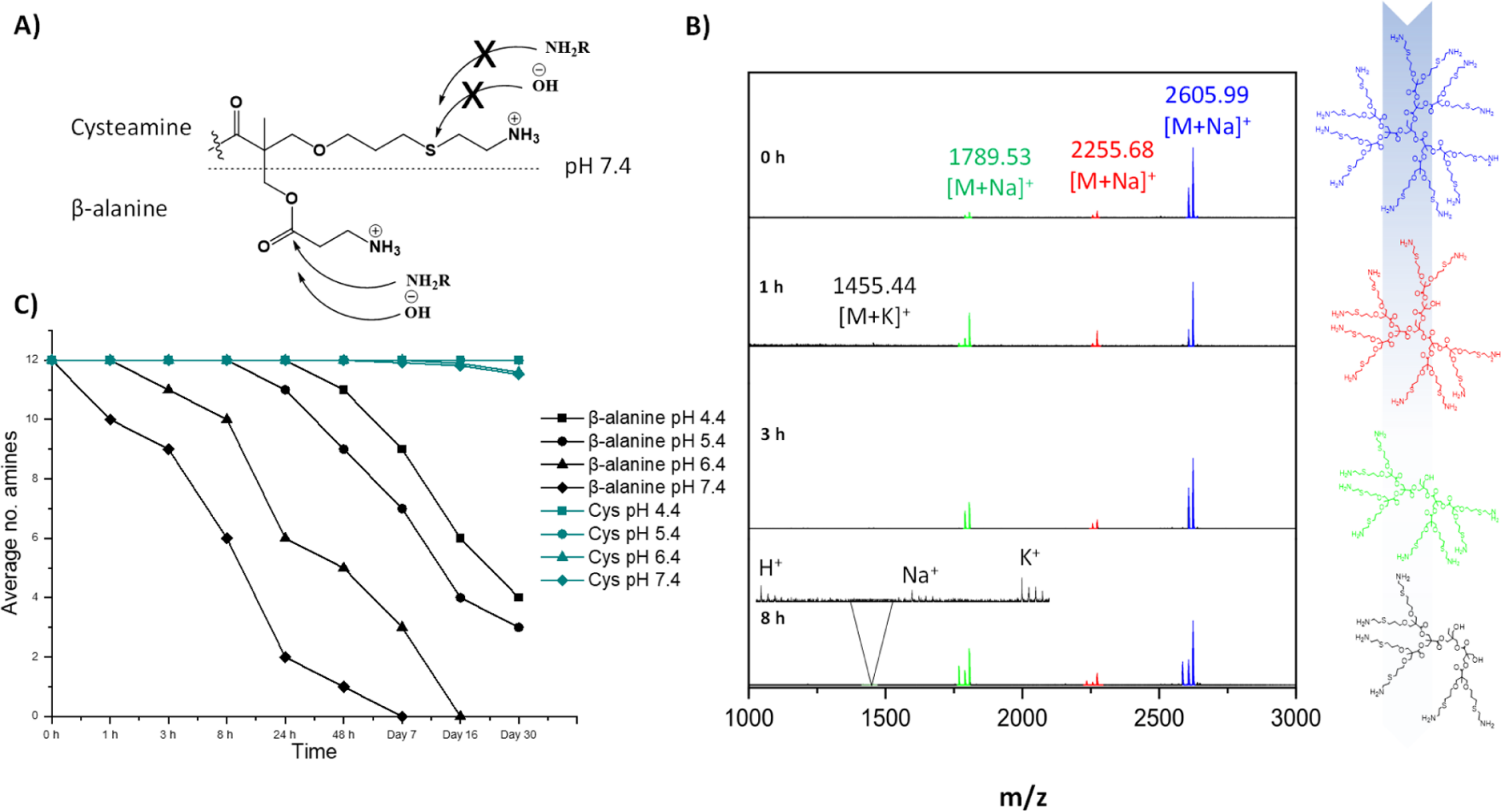
Degradation evaluation of the second-generation dendrimers followed by MALDI. (A) Proposed difference between lysis mechanism of β-alanine group compared cysteamine group. (B) Average number of amines present over a period of time for TMP-G2-[β-alanine]_12_ and TMP-G2-[Cys]_12_ at pH 4.4, 5.4, 6.4, and 7.4. (C) Stacked spectra for TMP-G2-[Cys]_12_ at pH = 13 and at different times (0, 1, 3, and 8 h).

Polymers including dendrimers designed for clinically oriented biomedical applications must have the ability to degrade in order to prevent bioaccumulation, which is associated with possible toxic effects. Previous reports have shown promising hydrolytic degradation processes of hydroxyl and β-alanine functional bis-MPA-based dendrimers at various pH and temperatures.^20,28^ Considering the envisioned increase in stability of the new dendrimers, the degradation rate was set as a function of pH and monitored by MALDI, as seen in Figure 2B.

As a reference, the degradation profile of previously reported TMP-G2-[β-alanine]_12_^28^ was monitored under parallel and identical conditions. The cysteamine dendrimer (TMP-G2-[Cys]_12_) showed increased hydrolytic stability compared to the β-alanine dendrimer. After 30 days, the cysteamine dendrimer was dominantly intact at all pHs (4.4–7.4). On average and based on calculated MALDI peak areas, after 1 month, 12 cysteamine groups were still present at low pHs 4.4 and 5.4 and 11 cysteamine groups at the peripheral at both pH 6.4 and 7.4. Meanwhile, for β-alanine dendrimers, after 30 days, only 4 and 3 amines were still present at pH 4.4 and pH 5.4, respectively, and complete loss of all amines was observed after 16 and 7 days at pH 6.4 and pH 7.4. These results confirm that pH has a strong effect on degradation and that bis-MPA dendrimers are generally more stable at low pHs. The increase in the degradation rate at higher pH can be explained by the increased nucleophilicity of the surrounding water and the dendrimer’s amine groups. This promotes lysis of the ester bond through hydrolysis or by intermolecular reactions with co-existing amine groups (Figure 2A). As postulated, the difference in the degradation rate and in aqueous different pHs could be manipulated by the bond representation at the peripheral layer of the polyester dendrimer. The β-alanine group is attached to the dendrimer via a hydrolysable ester bond, while the cysteamine group is via more stable thioether bond (Figure 2A). Importantly, the hydrolytic stability of the bis-MPA dendrimer was dramatically increased without the need of complete structural alternation but rather the addition of thioether at the peripheral was sufficient to protect the dendrimer from rapid degradation at physiological relevant pHs.

The depolymerization of β-alanine dendrimers proceeded by the gradual loss of all β-alanine groups was followed by the loss of bis-MPA moieties on the outer most layer. To showcase the mechanism at which the cysteamine dendrimer degrades, it was subjected to harsh conditions of pH 13 (Figure 2C). The study was monitored by MALDI at times 0, 1, 3, and 8 h. Interestingly, unlike the earlier published results on bis-MPA dendrimers, during depolymerization, the skeleton is compromised by the loss of cysteamine-functionalized bis-MPA units rather than the detachment of cysteamine groups. This is followed by additional dissection in which the skeleton is ruptured internally. Additionally, the flexible and extended representation of amines via cysteamine groups represented on TMP-G2-[Cys]_12_ was found to be incapable of attacking the interior ester bonds, neither inter- or intramolecularly. The new insight of degradation for these dendrimers, initiated by first access-to-internal esters, is indeed in stark contrast to the depolymerization process of traditional bis-MPA dendrimers.

### Biological Evaluation

Bis-MPA dendrimers decorated with cationic β-alanine showed great potential as antibacterial agents^27,28^ and were, therefore, used as a reference in the antibacterial evaluation of the stable bis-MPA cysteamine dendrimers. The MIC and MBC assays were used to evaluate from the first to third generation of cysteamine-functionalized dendrimers, as well as the previously reported β-alanine derivatives,^28^ toward Gram-negative *E. coli* and *P. aeruginosa* and Gram-positive *S. aureus* bacteria strains.

All cysteamine dendrimers showed higher antibacterial activity against all bacteria strains compared to β-alanine derivatives. For instance, the MIC for *E. coli*, *P. aeruginosa,* and *S. aureus* is roughly 100 times lower for TMP-G2-[Cys] than for_12_TMP-G2-[β-alanine]_12_. Considering that both systems have the same number of positive charges, the drastic difference observed could be explained by the high degradation presented by β-alanine dendrimers compared to cysteamine derivatives. The antibacterial activity is mainly provided by the peripheral cationic groups and, therefore, correlated with the stability of the system. From the degradation study (Figure 2), after 24 h at physiological pH 7.4, only around 20 % of the cationic functionalities remained attached to the skeleton in β-alanine dendrimers, whereas the increased stability of cysteamine dendrimers under the same conditions ensured the presence of a fully functionalized dendrimer. Additionally, for both families of dendrimers, an increase in the dendritic generation involved an improvement in the antibacterial activity against all bacteria strains. Interestingly, our systems were generally found to be more active against *E. coli* and *P. aeruginosa*, in spite of the well-known higher resistance offered by Gram-negative bacteria compared to Gram-positive ones.^32^ The observed difference in activity against Gram-negative and Gram-positive strains was with the exception of TMP-G1-[Cys]_6_ and TMP-G3-[β-alanine]_24_, where less activity toward Gram-negative *P. aeruginosa* was seen. Additionally, taking into account the number of functional groups per dendritic molecule, the MIC and MBC values were calculated based on the concentration of ammonium groups. As stated above, first generation dendrimers showed the lowest antibacterial activity in both families. However, the second and third generation cysteamine dendrimers presented the same MIC value against *E. coli* (19.2 μM), indicating an increase in the dendritic generation did not drastically change the antibacterial activity.

**Figure 3:**
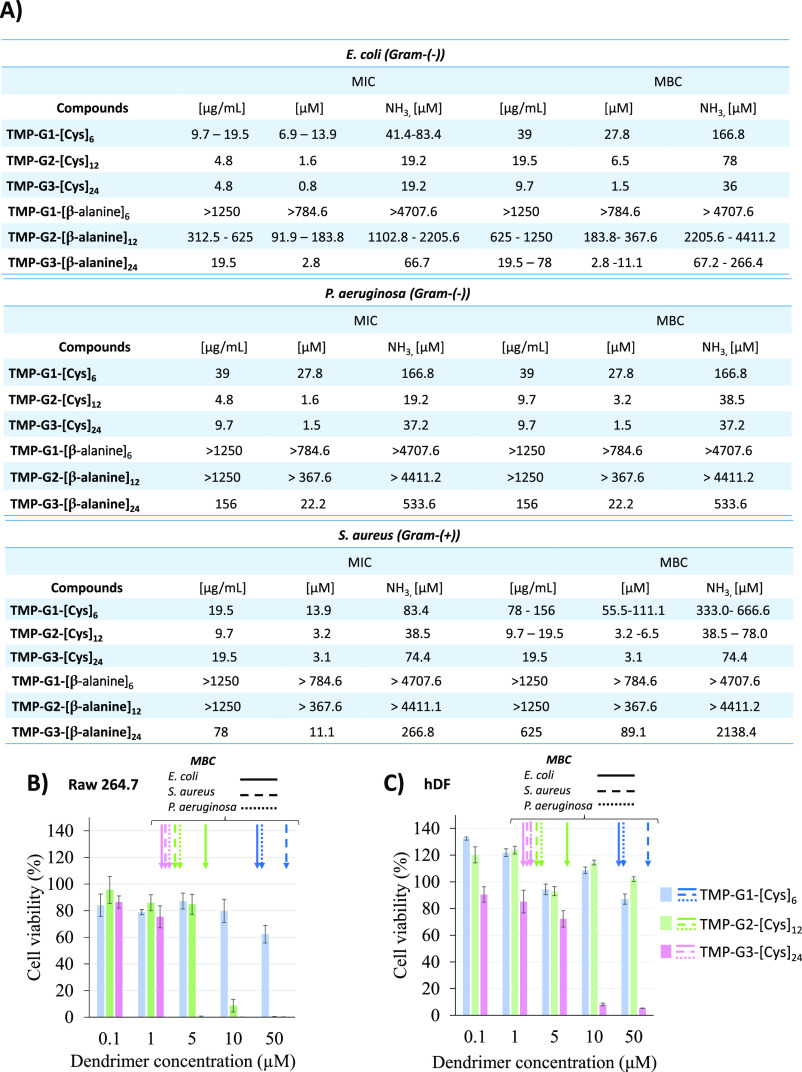
(A) MIC and MBC for the new family of cysteamine-functionalized dendrimers as well as the previously reported β-alanine cationic dendrimers. (B,C) AlamarBlue assay for the cytotoxicity evaluation of the cationic dendrimers toward (B) monocytes/macrophages like cells (Raw 264.7) and (C) hDFs after incubation for 24 h.

In spite of the promising antibacterial activity provided by positive charges, one of the main drawbacks in the use of cationic systems for biomedical applications is their inherent toxicity attributed to the interaction of the surface cationic charge with negatively charged biological membranes in vivo.^33^ For a better understanding about the toxicity of the new family of stable cationic bis-MPA dendrimers, a complete screening of cell viability has been conducted against hDF and Raw 264.7 cells by using concentrations ranged from 0.1 to 50 μM after 24 h of incubation. The results represented in Figure 3 show a generation and dose-dependent effect. The first-generation dendrimer (TMP-G1-[Cys]_6_), with six cationic charges, did not present toxicity in fibroblasts at any of the tested concentrations (Figure 3B) and only at the highest concentration toward monocytes (Figure 3A). By doubling the number of positive charges for generation two (TMP-G2-[Cys]_12_), no significant differences could be appreciated toward fibroblasts (Figure 3B). However, the toxicity of the second-generation derivative is more evident for Raw 264.7 cells, with a viability less than 20 % at 10 μM of dendrimers. Finally, the third-generation dendrimer (TMP-G3-[Cys]_24_), with 24 functional groups, is the most toxic and could be only used up to 1 and 5 μM, for monocytes and fibroblasts, respectively. The comparison with previously reported cell viability evaluations for β-alanine dendritic derivatives shows that an increase in the hydrolytic stability does not significantly alter the toxicity of the final molecule. The only variation has been observed for generation two, where the derivative with β-alanine (TMP-G2-[β-alanine]_12_) was not toxic at any of the tested concentrations in any of the tested cell lines,^34^ whereas after an increased stability, the new system (TMP-G2-[Cys]_12_) is more toxic for raw cells. In the evaluation of a potential antibacterial compound, it is imperative to consider the toxicity at specific concentrations at which antibacterial activity is observed. As shown in Figure 3, cysteamine dendrimers exhibit no toxicity at concentrations at which they are active against all three strains with the exception of TMP-G1-[Cys]_6_ against *S. aureus* and *P. aeruginosa* toward the raw 264.7 cell line. Overall, TMP-G2-[Cys]_12_ stands out as the most promising candidate as an antibacterial agent exhibiting low toxicity at the concentration at which it is active against bacteria.

### Structural Diversity

**Figure 4:**
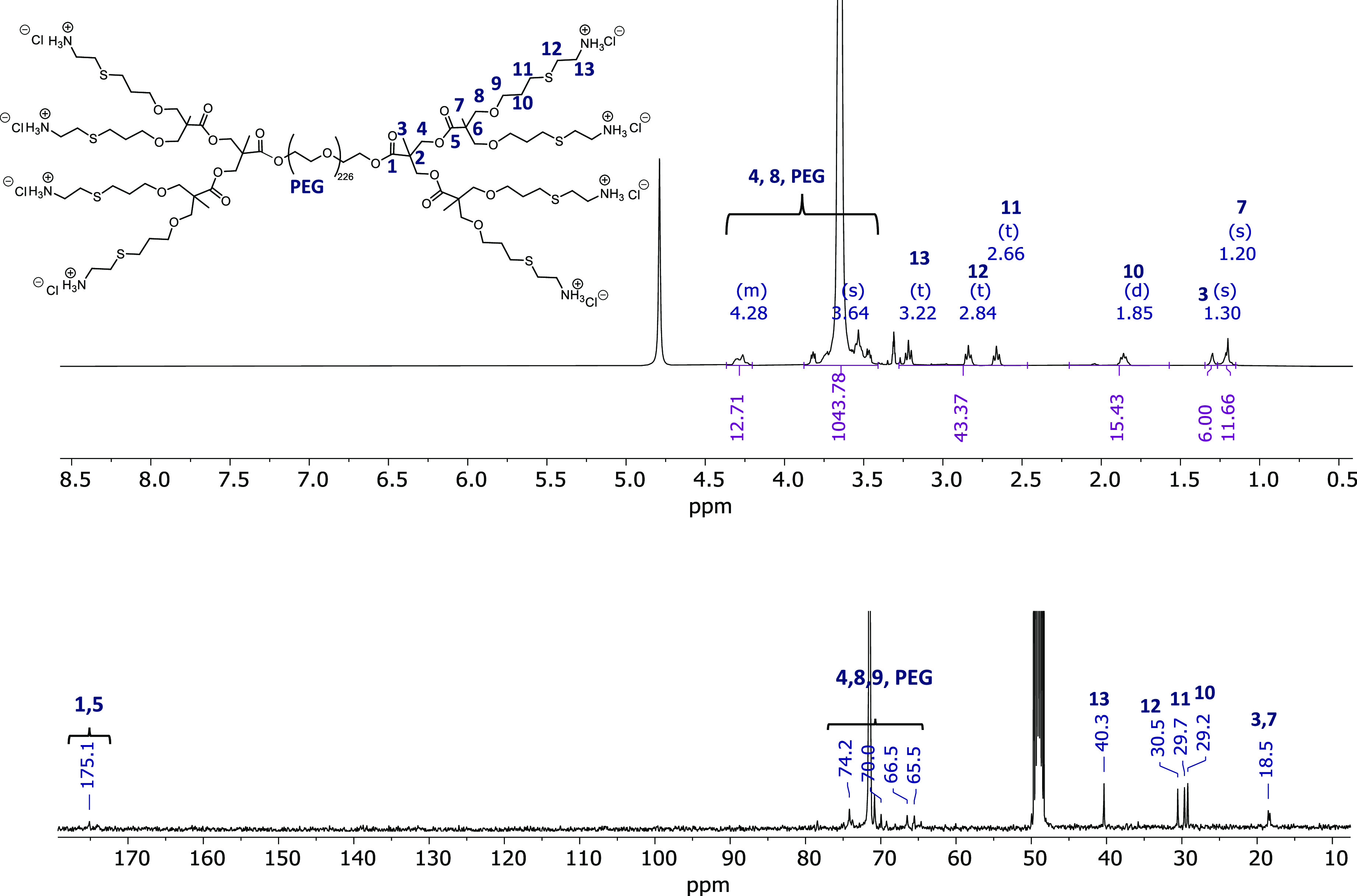
^1^H and ^13^C NMR spectra of the PEG10k-G2-[Cys]_8_ in CD_3_OD.

Satisfied with the chemical stability and biological performance of the cysteamine-functionalized dendrimers, we expanded the structural library by first targeting the synthesis of DLDs using similar synthetic protocols as for the dendrimers. The DLDs based on a bis-MPA configuration are considered as amphiphilic “bow-tied” systems that often contain a water-soluble PEG chain and two bis-MPA hydrophobic dendrons. Starting from previously described PEG10k-DLD precursors with hydroxyl functionalities,^35^ alkenes were introduced though esterification reactions using BAPA anhydrides. Subsequently, the alkenes were reacted with cysteamine hydrochloride through TEC click chemistry. The monitoring of these reactions was mainly conducted using ^1^H- and ^13^C-NMR (Figure 4), as well as SEC for the BAPA intermediates (Figure S13). DLDs ranged from generation one up to generation three, with 4, 8, and 16 functionalities were obtained and purified by using Sephadex G-10 isolating sticky yellow solids with yields between 40 and 70 %. β-Alanine-functionalized DLDs have been previously evaluated as dendritic components in antibacterial hydrogel formulations and for a potential application toward surgical site infections.^35^ The inclusion of cysteamine as a source of positives charges into DLD structures makes them a hydrolytic stable option to be further evaluated as precursors to generate antibacterial hydrogels.

### Post-Functionalization of Dendrimers

**Figure 5:**
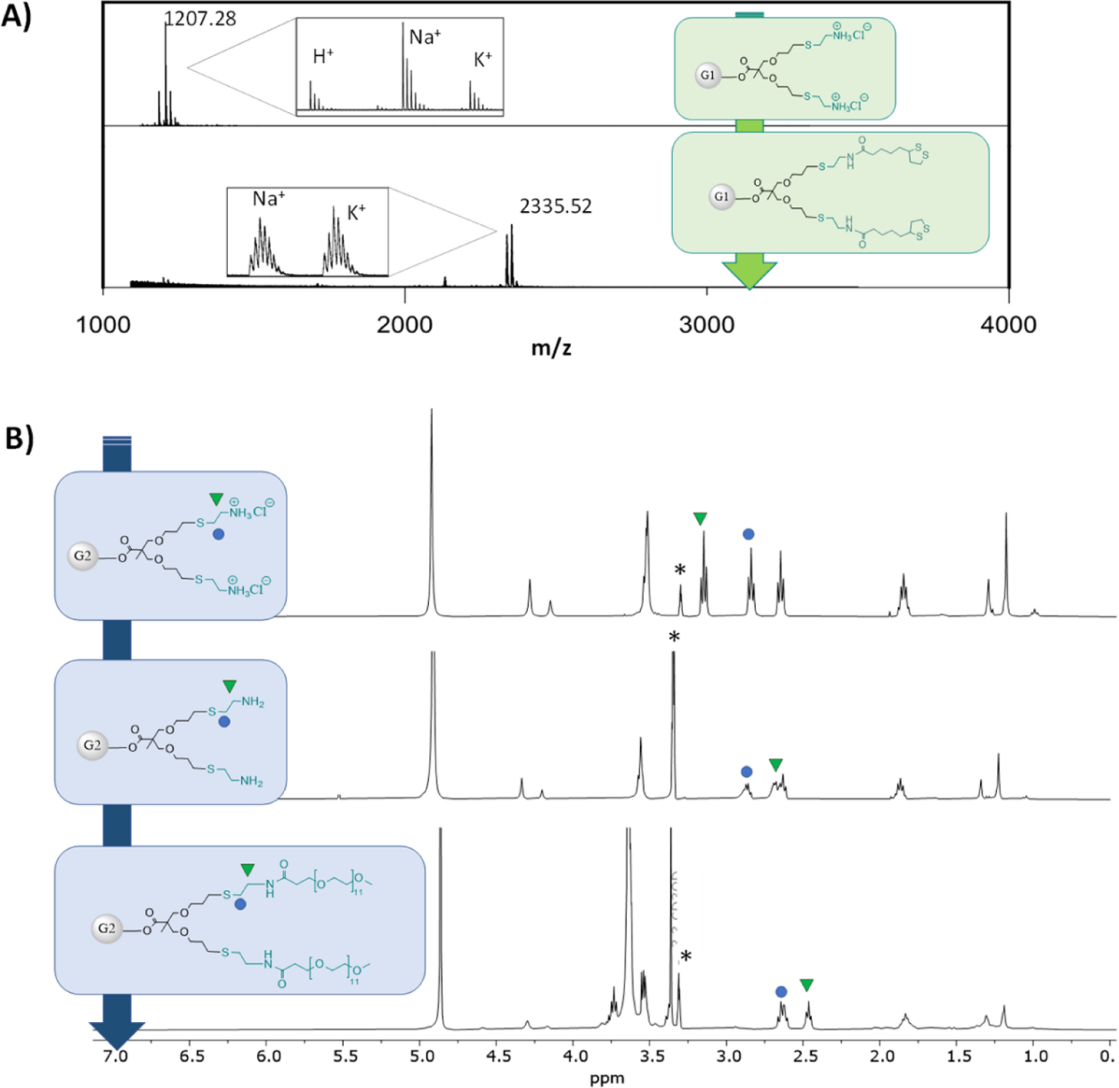
Post-functionalization of cationic dendrimers with lipoic acid mPEG. (A) MALDI-TOF for the first-generation dendrimers (TMP-G1-[Cys]_6_ and TMP-G1-[Cys-lipoic acid]_6_ with theoretical values of [M + Na]_Theo_^+^: 1207.59 Da and [M + Na]_Theo_^+^: 2335.79 Da, respectively). (B) ^1^H-NMR spectra for the second-generation dendrimers (TMP-G2-[Cys]_12_ (charged and neutral) and TMP-G2-[Cys-mPEG_11_]_12_) in CD_3_OD (*).

In another context, the post-functionalization via amidation reactions of the cysteamine functional dendrimers was investigated. Here, lipoic acid and PEGs were considered important substituents that find use in biomedical applications. For the former, lipoic acid was sought out as an interesting substituent having promising antioxidant properties. The lipoic acid decreases the levels of reactive oxygen species due to the disulfide bond with reductive capabilities. This molecule has played an important role in the fight against diseases associated with a redox imbalance, such as diabetes and cardiovascular diseases.^36^ Additionally, the rationale behind PEGylation of a biologically active molecule is the potential for improving the therapeutic properties, for instance, enhancing aqueous solubility and prolonging blood circulation time in vivo.^37^ As a result, the TMP-G1-[Cys]_6_ and TMP-G2-[Cys]_12_ were post-functionalized through amidation reactions with lipoic acid (Figure 5A) and mPEG11-COOH (Figure 5B), respectively. The efficient inclusion of both functionalities through the amidation reaction was ensured by neutralizing both dendritic generations with NaHCO_3_ until around pH 8 and monitored by ^1^H-NMR. For functionalization with lipoic acid, CDI was used as a coupling agent. The activation of the lipoic acid was completed after 1 h of reaction using DCM as a solvent and at room temperature. Subsequently, the activated acid was added to a suspension of the neutral dendrimer in DCM that dissolves as the reaction progresses at 50 °C. This reaction was monitored through MALDI until full conversion. Meanwhile, DCC was used to activate the mPEG11 acid to form the corresponding anhydride using DCM as the solvent. ^13^C-NMR was used to confirm the completion of the overnight reaction, before adding to a solution of the neutral dendrimer. To corroborate the full functionalization, MALDI as well as ^1^H-NMR were utilized. As shown in Figure 5B, the shift of the methylene protons in the cysteamine moiety to lower ppm confirmed the reaction completion.

## Conclusions

Bis-MPA-based dendrimers functionalized with cysteamine from generation one to three were successfully synthesized using robust and facile synthetic strategies. The modification of the outer layer through TEC comprising thioether bonds and displaying cysteamine functionalities was found to increase hydrolytic stability at various physiologically relevant pHs compared to previously reported β-alanine derivatives. Interestingly, an alternative depolymerization mechanism for these new dendrimers was observed. Unlike β-alanine-functionalized bis-MPA dendrimers, the loss of the first functionalized bis-MPA moiety at the periphery was followed by an inner layer bis-MPA group rather than a gradual loss at the surface layer. The increased stability was accompanied by an improvement in antibacterial activity against both Gram-positive and Gram-negative planktonic bacteria strains. Considering the high toxicity associated with cationic systems, surprisingly, this novel family of dendrimers is not toxic at the concentration at which it is active against bacteria. The second-generation dendrimer could be considered as the most promising candidate for a biomedical application providing the highest benefits and reducing the efforts and costs associated with the production. Additionally, the reaction strategy versatility was demonstrated by the successful synthesis of the first, second, and third generation of cationic PEG10k-DLDs. Finally, the presence of peripheral amines opened up a range of possibilities for using these systems as precursors for further functionalization with a wide variety of biologically interesting molecules, as showcased with the PEGylation and inclusion of lipoic acid.

## Supporting Information Available

Synthetic protocols of all dendrimers and DLDs, ^1^H and ^13^C NMR spectra, MALDI–TOF spectra, SEC chromatographs, and degradation evaluation of generation two dendrimers monitored MALDI–TOF (PDF)

## References

[ref1] Carlmark A.; Hawker C.; Hult A.; Malkoch M. New methodologies in the construction of dendritic materials. Chem. Soc. Rev. 2009, 38, 352–362. 10.1039/b711745k. 19169453

[ref2] Carlmark A.; Malmström E.; Malkoch M. Dendritic architectures based on bis-MPA: functional polymeric scaffolds for application-driven research. Chem. Soc. Rev. 2013, 42, 5858–5879. 10.1039/c3cs60101c. 23628841

[ref3] Malkoch M.; Malmström E.; Nyström A. Dendrimers: properties and applications. Polymer Science: A Comprehensive Reference; Elsevier, 2012; Vol. 6, pp 113–176.

[ref4] Walter M. V.; Malkoch M. Simplifying the synthesis of dendrimers: accelerated approaches. Chem. Soc. Rev. 2012, 41, 4593–4609. 10.1039/c2cs35062a. 22592560

[ref5] Tomalia D. A.; Baker H.; Dewald J.; Hall M.; Kallos G.; Martin S.; Roeck J.; Ryder J.; Smith P. A new class of polymers: starburst-dendritic macromolecules. Polym. J. 1985, 17, 117–132. 10.1295/polymj.17.117.

[ref6] Newkome G. R.; Yao Z.; Baker G. R.; Gupta V. K. Micelles. Part 1. Cascade molecules: a new approach to micelles. A [27]-arborol. J. Org. Chem. 1985, 50, 2003–2004. 10.1021/jo00211a052.

[ref7] Kaczorowska A.; Malinga-Drozd M.; Kałas W.; Kopaczyńska M.; Wołowiec S.; Borowska K. Biotin-Containing Third Generation Glucoheptoamidated Polyamidoamine Dendrimer for 5-Aminolevulinic Acid Delivery System. Int. J. Mol. Sci. 2021, 22, 1982 10.3390/ijms22041982. 33671436 PMC7922973

[ref8] Swanson S. D.; Kukowska-Latallo J. F.; Patri A. K.; Chen C.; Ge S.; Cao Z.; Kotlyar A.; East A. T.; Baker J. R. Targeted gadolinium-loaded dendrimer nanoparticles for tumor-specific magnetic resonance contrast enhancement. Int. J. Nanomed. 2008, 3, 201–210. PMC2527674 18686779

[ref9] Shi X.; Wang S. H.; Swanson S. D.; Ge S.; Cao Z.; Van Antwerp M. E.; Landmark K. J.; Baker J. R. Jr Dendrimer-functionalized shell-crosslinked iron oxide nanoparticles for in-vivo magnetic resonance imaging of tumors. Adv. Mater. 2008, 20, 1671–1678. 10.1002/adma.200702770.

[ref10] Zhou J.; Wu J.; Hafdi N.; Behr J.-P.; Erbacher P.; Peng L. PAMAM dendrimers for efficient siRNA delivery and potent gene silencing. Chem. Commun. 2006, 22, 2362–2364. 10.1039/b601381c. 16733580

[ref11] Jain K.; Kesharwani P.; Gupta U.; Jain N. Dendrimer toxicity: Let’s meet the challenge. Int. J. Pharm. 2010, 394, 122–42. 10.1016/j.ijpharm.2010.04.027. 20433913

[ref12] Malik N.; Wiwattanapatapee R.; Klopsch R.; Lorenz K.; Frey H.; Weener J.; Meijer E.; Paulus W.; Duncan R. Dendrimers:: Relationship between structure and biocompatibility in vitro, and preliminary studies on the biodistribution of 125I-labelled polyamidoamine dendrimers in vivo. J. Controlled Release 2000, 65, 133 10.1016/s0168-3659(99)00246-1. 10699277

[ref13] Mukherjee S. P.; Davoren M.; Byrne H. J. In vitro mammalian cytotoxicological study of PAMAM dendrimers–towards quantitative structure activity relationships. Toxicol. Vitro 2010, 24, 169–177. 10.1016/j.tiv.2009.09.014. 19778601

[ref14] Mukherjee S. P.; Lyng F. M.; Garcia A.; Davoren M.; Byrne H. J. Mechanistic studies of in vitro cytotoxicity of poly (amidoamine) dendrimers in mammalian cells. Toxicol. Appl. Pharmacol. 2010, 248, 259–268. 10.1016/j.taap.2010.08.016. 20736030

[ref15] Hong S.; Leroueil P. R.; Janus E. K.; Peters J. L.; Kober M.-M.; Islam M. T.; Orr B. G.; Baker J. R. Jr; Holl M. M. B. Interaction of polycationic polymers with supported lipid bilayers and cells: nanoscale hole formation and enhanced membrane permeability. Bioconjugate Chem. 2006, 17, 728–734. 10.1021/bc060077y. 16704211

[ref16] Wang R. E.; Costanza F.; Niu Y.; Wu H.; Hu Y.; Hang W.; Sun Y.; Cai J. Development of self-immolative dendrimers for drug delivery and sensing. J. Controlled Release 2012, 159, 154–163. 10.1016/j.jconrel.2011.11.032. 22155555

[ref17] García-Gallego S.; Hult D.; Olsson J. V.; Malkoch M. Fluoride-Promoted Esterification with Imidazolide-Activated Compounds: A Modular and Sustainable Approach to Dendrimers. Angew. Chem., Int. Ed. 2015, 54, 2416–2419. 10.1002/anie.201411370. 25605503

[ref18] Gillies E. R.; Dy E.; Fréchet J. M.; Szoka F. C. Biological evaluation of polyester dendrimer: poly (ethylene oxide)“bow-tie” hybrids with tunable molecular weight and architecture. Mol. Pharm. 2005, 2, 129–138. 10.1021/mp049886u. 15804187

[ref19] De Jesús O. L. P.; Ihre H. R.; Gagne L.; Fréchet J. M.; Szoka F. C. Polyester dendritic systems for drug delivery applications: in vitro and in vivo evaluation. Bioconjugate Chem. 2002, 13, 453–461. 10.1021/bc010103m. 12009933

[ref20] Feliu N.; Walter M. V.; Montañez M. I.; Kunzmann A.; Hult A.; Nyström A.; Malkoch M.; Fadeel B. Stability and biocompatibility of a library of polyester dendrimers in comparison to polyamidoamine dendrimers. Biomaterials 2012, 33, 1970–1981. 10.1016/j.biomaterials.2011.11.054. 22177621

[ref21] Zhang Y.; Mesa-Antunez P.; Fortuin L.; Andrén O. C.; Malkoch M. Degradable High Molecular Weight Monodisperse Dendritic Poly (ethylene glycols). Biomacromolecules 2020, 21, 4294–4301. 10.1021/acs.biomac.0c01089. 32845125

[ref22] Lichter J. A.; Van Vliet K. J.; Rubner M. F. Design of antibacterial surfaces and interfaces: polyelectrolyte multilayers as a multifunctional platform. Macromolecules 2009, 42, 8573–8586. 10.1021/ma901356s.

[ref23] Holmes A. M.; Heylings J. R.; Wan K.-W.; Moss G. P. Antimicrobial efficacy and mechanism of action of poly (amidoamine)(PAMAM) dendrimers against opportunistic pathogens. Int. J. Antimicrob. Agents 2019, 53, 500–507. 10.1016/j.ijantimicag.2018.12.012. 30599243

[ref24] Serri A.; Mahboubi A.; Zarghi A.; Moghimi H. R. PAMAM-dendrimer enhanced antibacterial effect of vancomycin hydrochloride against gram-negative bacteria. J. Pharm. Pharm. Sci. 2019, 22, 10–21. 10.18433/jpps29659. 30589641

[ref25] Murata H.; Koepsel R. R.; Matyjaszewski K.; Russell A. J. Permanent, non-leaching antibacterial surfaces—2: How high density cationic surfaces kill bacterial cells. Biomaterials 2007, 28, 4870–4879. 10.1016/j.biomaterials.2007.06.012. 17706762

[ref26] Milović N. M.; Wang J.; Lewis K.; Klibanov A. M. Immobilized N-alkylated polyethylenimine avidly kills bacteria by rupturing cell membranes with no resistance developed. Biotechnol. Bioeng. 2005, 90, 715–22. 10.1002/bit.20454. 15803464

[ref27] Fan Y.; Namata F.; Erlandsson J.; Zhang Y.; Wågberg L.; Malkoch M. Self-assembled polyester dendrimer/cellulose nanofibril hydrogels with extraordinary antibacterial activity. Pharmaceutics 2020, 12, 1139 10.3390/pharmaceutics12121139. 33255607 PMC7761394

[ref28] Stenström P.; Hjorth E.; Zhang Y.; Andrén O. C.; Guette-Marquet S.; Schultzberg M.; Malkoch M. Synthesis and in vitro evaluation of monodisperse amino-functional polyester dendrimers with rapid degradability and antibacterial properties. Biomacromolecules 2017, 18, 4323–4330. 29131611 10.1021/acs.biomac.7b01364

[ref29] García-Gallego S.; Nyström A. M.; Malkoch M. Chemistry of multifunctional polymers based on bis-MPA and their cutting-edge applications. Prog. Polym. Sci. 2015, 48, 85–110. 10.1016/j.progpolymsci.2015.04.006.

[ref30] Malkoch M.; Malmström E.; Hult A. Rapid and efficient synthesis of aliphatic ester dendrons and dendrimers. Macromolecules 2002, 35, 8307–8314. 10.1021/ma0205360.

[ref31] Montañez M. I.; Campos L. M.; Antoni P.; Hed Y.; Walter M. V.; Krull B. T.; Khan A.; Hult A.; Hawker C. J.; Malkoch M. Accelerated Growth of Dendrimers via Thiol–Ene and Esterification Reactions. Macromolecules 2010, 43, 6004–6013. 10.1021/ma1009935.

[ref32] Breijyeh Z.; Jubeh B.; Karaman R. Resistance of Gram-Negative Bacteria to Current Antibacterial Agents and Approaches to Resolve It. Molecules 2020, 25, 1340 10.3390/molecules25061340. 32187986 PMC7144564

[ref33] Jain K.; Kesharwani P.; Gupta U.; Jain N. K. Dendrimer toxicity: Let’s meet the challenge. Int. J. Pharm. 2010, 394, 122–42. 10.1016/j.ijpharm.2010.04.027. 20433913

[ref34] Stenström P.; Hjorth E.; Zhang Y.; Andrén O. C. J.; Guette-Marquet S.; Schultzberg M.; Malkoch M. Synthesis and in Vitro Evaluation of Monodisperse Amino-Functional Polyester Dendrimers with Rapid Degradability and Antibacterial Properties. Biomacromolecules 2017, 18, 4323–4330. 10.1021/acs.biomac.7b01364. 29131611

[ref35] Andrén O. C.; Ingverud T.; Hult D.; Håkansson J.; Bogestål Y.; Caous J. S.; Blom K.; Zhang Y.; Andersson T.; Pedersen E.; Björn C.; Löwenhielm P.; Malkoch M. Antibiotic-Free Cationic Dendritic Hydrogels as Surgical-Site-Infection-Inhibiting Coatings. Adv. Healthcare Mater. 2019, 8, 1801619 10.1002/adhm.201801619. 30735288

[ref36] Rochette L.; Ghibu S.; Richard C.; Zeller M.; Cottin Y.; Vergely C. Direct and indirect antioxidant properties of α-lipoic acid and therapeutic potential. Mol. Nutr. Food Res. 2013, 57, 114–125. 10.1002/mnfr.201200608. 23293044

[ref37] Turecek P. L.; Bossard M. J.; Schoetens F.; Ivens I. A. PEGylation of biopharmaceuticals: a review of chemistry and nonclinical safety information of approved drugs. J. Pharm. Sci. 2016, 105, 460–475. 10.1016/j.xphs.2015.11.015. 26869412

