# Supplementary material for: Synthesis and Characterization of Amino-Functional Polyester Dendrimers Based On Bis-MPA with Enhanced Hydrolytic Stability and Inherent Antibacterial Properties
Source: Biomacromolecules. 2023 Jan 23;24(2):858–67. doi: 10.1021/acs.biomac.2c01286 (PMC9930107; doi:10.1021/acs.biomac.2c01286)
Supplement: Supplementary file 1 — bm2c01286_si_001.pdf [file bm2c01286_si_001.pdf]

Electronic Supporting Information

Synthesis and Characterization of Amino-Functional Polyester Dendrimers based on bis-MPA with Enhanced Hydrolytic Stability and Inherent Antibacterial Properties

Faridah Namata [1, ‡], Natalia Sanz del Olmo [1, ‡], Noemi Molina [1] and Michael Malkoch [1,].*

[1] Department of Fibre and Polymer Technology, KTH Royal Institute of Technology, Teknikringen 56-68, 100 44, Stockholm, Sweden.

[*] Author to whom correspondence should be addressed.

[‡] These authors contributed to this work equally.

Corresponding author: Prof. Michael Malkoch

Contact info: School of Engineering Sciences in Chemistry, Biotechnology and Health; Department of Fibre and Polymer Technology; Division of Coating Technology Teknikringen 48, SE-10044, Stockholm

Fax: (+) 46 (0)8 790 82 83

E-mail: malkoch@kth.se

Table of contents

Synthesis Protocols 3

Figures..11

Figure S1. ^{1}H and ^{13}C NMR spectra of the dendrimer TMP-G1-[ene]$_6$ in CD_3OD.............................11

Figure S2. ^{1}H and ^{13}C NMR spectra of the dendrimer TMP-G2-[ene]$_{12}$ in CD_3OD. 11

Figure S3. ^{1}H and ^{13}C NMR spectra of the dendrimer TMP-G3-[ene]$_{24}$ in CD_3OD...........................12

Figure S4. SEC overlay of TMP-G1-[ene]$_6$, TMP-G2-[ene]$_{12}$ and TMP-G3-[ene]$_{24}$ 12

Figure S5. ^{1}H and ^{13}C NMR spectra of the dendrimer TMP-G1-[Cys]$_6$ in CD_3OD. 13

Figure S6. ^{1}H and ^{13}C NMR spectra of the dendrimer TMP-G2-[Cys]$_{12}$ in CD_3OD...........................13

Figure S7. ^{1}H and ^{13}C NMR spectra of the dendrimer TMP-G3-[Cys]$_{24}$ in CD_3OD...........................14

Figure S8. MALDI-ToF spectra of the dendrimers TMP-G1-[ene]$_6$, TMP-G2-[ene]$_{12}$, TMP-G3-[ene]$_{24}$, TMP-G1-[Cys]$_6$, TMP-G2-[Cys]$_{12}$ and TMP-G3-[Cys]$_{24}$...14

Figure S9. Degradation evaluation through MALDI-ToF of the dendrimers a) TMP-G2-[β-alanine]$_{12}$ and b) TMP-G2-[Cys]$_{12}$ at different pH and times. 15

Figure S10. ^{1}H and ^{13}C NMR spectra of the PEG10K-G1-[ene]$_4$ in $CDCl_3$.......................................15

Figure S11. ^{1}H and ^{13}C NMR spectra of the PEG10K-G2-[ene]$_8$ in $CDCl_3$. 16

Figure S12. ^{1}H and ^{13}C NMR spectra of the PEG10K-G3-[ene]$_{16}$ (9) in $CDCl_3$. 16

Figure S13. SEC overlay of PEG10K-G1-[ene]$_4$, PEG10K-G2-[ene]$_8$ and PEG10K-G3-[ene]$_{16}$........17

Figure S14. ^{1}H and ^{13}C NMR spectra of the PEG10K-G1-[Cys]$_4$ in CD_3OD. 17

Figure S15. ^{1}H and ^{13}C NMR spectra of the PEG10K-G2-[Cys]$_8$ in CD_3OD......................................18

Figure S16. ^{1}H and ^{13}C NMR spectra of the PEG10K-G3-[Cys]$_{16}$ in CD_3OD. 18

Figure S17. ^{1}H NMR spectrum of the TMP-G1-[Cys-lipoic acid]$_6$ 19

Figure S18. ^{1}H NMR spectrum of the TMP-G2-[Cys-mPEG$_{11}$]$_{12}$..19

References..20

Synthesis Protocols

A

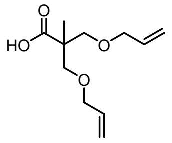

B

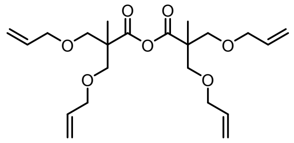

C

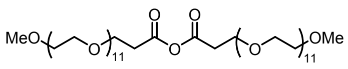

D

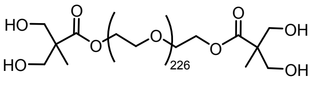

E

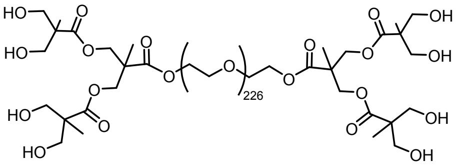

F

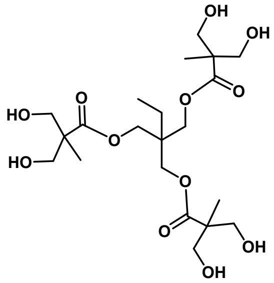

G

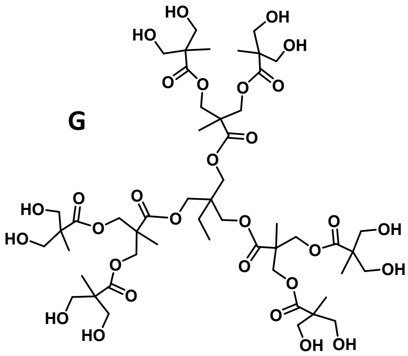

The following precursors were prepared as previously published: A) BAPA acid[1], B) BAPA anhydride[1], C) $mPEG_{11}$ anhydride[4], hydroxyl functional dendritic PEG D) PEG10k-G1-$[OH]_4$ and E) PEG10k-G2-$[OH]_8$)[3] and .hydroxyl functional Bis-MPA dendrimers F) TMP-G1-$[OH]_6$ and G) TMP-G2-$[OH]_{12}$)[2].

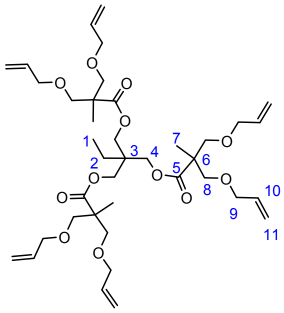

TMP-G1-[ene]$_6$. CDI (7.6 g, 46.7 mmol) was slowly added over a solution of BAPA acid (10.0 g, 46.7 mmol) in EtOAc. After 1 hour of stirring at room temperature, the crude mixture is added to a solution of the hydroxy-functional derivative TMP (1.74 g, 12.9 mmol) and CsF (0.2 eq/OH) in EtOAc. The mixture was kept under stirring at 50 ºC overnight. The reaction was monitored by NMR and MALDI-TOF. Upon completion, the mixture was allowed to cool to room temperature, and the excess of imidazolide-activated acid was quenched by stirring with water. The mixture was then diluted with EtOAc and washed repeatedly with aqueous solutions of 10 % $NaHCO_3$ and 10 % $NaHSO_4$, and once with brine before being dried with $MgSO_4$, filtered and evaporated. Dendrimer TMP-G1-$[ene]_6$ was obtained as a yellow viscous oil upon concentration *in vacuo* (9.0 g, 96 %). ^{1}H-NMR (400 MHz, CD_3OD) δ/ppm: 5.87 (6H, ddt, J = 6, 11, 17 Hz, H10), 5.25 (6H, dq, J = 2, 17 Hz, H11a), 5.14 (6H, dq, J = 2, 11 Hz, H11b), 4.07 (6H, s, H4), 3.96 (12H, dt, J = 2, 6 Hz, H9), 3.54 (12H, q, J = 9 Hz, H8), 1.51 (2H, q, J = 8 Hz, H2), 1.21 (9H, s, H7), 0.91 (3H, t, J = 8 Hz, H1). ^{13}C-NMR (101 MHz, CD_3OD) δ/ppm: 175.5 (C5), 136.1 (C10), 117.1 (C11), 74.0 (C8), 73.3 (C9), 64.5 (C4), 49.8 (C6), 42.9 (C3), 23.9 (C2), 18.5 (C7), 7.8 (C1). MALDI-TOF: $[M+Na]^+_{Theo}$: 745.410 Da, $[M+Na]^+_{Exp}$: 745.57 Da,

$[M+K]^+_{Theo}$: 761.518 Da, $[M+K]^+_{Exp}$: 761.56 Da. SEC (DMF) M_n =821.38 g mol^{-1}, M_w= 833.31 g mol^{-1}, Đ = 1.01.

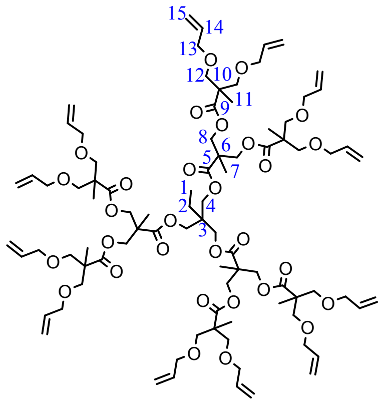

TMP-G2-[ene]$_{12}$. CDI (10.2 g, 63.1 mmol) was slowly added over a solution of BAPA (13.5 g, 63.1 mmol) in EtOAc. After 1 h of stirring at room temperature the crude mixture was added to a solution of TMP-G1-OH (4.22 g, 8.75 mmol) and CsF (0.2 eq/OH) in EtOAc. The mixture was kept under stirring at 50 ºC overnight. The reaction was monitored by NMR and MALDI-TOF. Upon completion the mixture was allowed to cool to room temperature, and the excess of imidazolide-activated acid was quenched by stirring with water. The mixture was then diluted with EtOAc and washed repeatedly with aqueous solutions of 10 % $NaHCO_3$ and 10% $NaHSO_4$, and once with brine before being dried with $MgSO_4$, filtered and evaporated. TMP-G2-[ene]$_{12}$ was obtained as a pale-yellow oil upon concentration (13.5 g, 93 %). ^{1}H-NMR (400 MHz, CD_3OD) δ/ppm: 5.88 (12H, ddt, J = 6, 11, 17 Hz, H14), 5.25 (12H, dq, J = 2, 17 Hz, H15a), 5.14 (12 H, dq, J = 2, 11 Hz, H15b), 4.29 (12H, s, H8), 4.13 (6H, s, H4), 3.96 (24H, dt, J = 2, 6 Hz, H13), 3.60 – 3.46 (24H, m, H12), 1.56 (2H, m, H2), 1.27 (9H, s, H7), 1.20 (18H, s, H11), 0.96 (3H, t, J = 8 Hz, H1). ^{13}C-NMR (101 MHz, CD_3OD) δ/ppm: 175.1 (C9), 173.5 (C5), 136.1 (C14), 117.1 (C15), 73.4 (C12), 73.3 (C13), 66.1 (C8), 65.0 (C4), 49.8 (C10), 48.2 (C6), 42.8 (C3), 24.1 (C2), 18.5 (C11), 18.3 (C7), 8.2 (C1). MALDI-TOF: $[M+Na]^+_{Theo}$: 1681.89 Da, $[M+Na]^+_{Exp}$: 1681.94 Da, $[M+K]^+_{Theo}$: 1698.00 Da, $[M+K]^+_{Exp}$: 1697.91 Da. SEC (DMF) M_n =1698.7 g mol-1, M_w= 1723.0 g mol-1, Đ = 1.01.

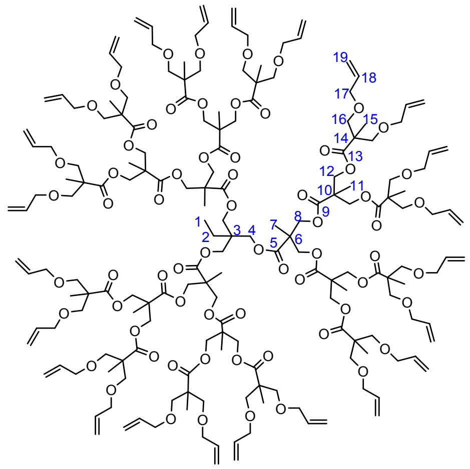

TMP-G3-[ene]$_{24}$. In a RBF equipped with a magnetic stirrer TMP-G2-OH (10.6 g, 8.98 mmol) was dissolved in pyridine (72 mL, 0.89 mol) and DCM. DMAP (2.64 g, 21.57 mmol) was added and under vigorous stirring BAPA anhydride (88.6 g, 216 mmol) was slowly added as a powder. The reaction was allowed to proceed for 16 hours and the progression confirmed with NMR and MALDI. Once completion, the crude reaction mixture was washed repeatedly with aqueous solutions of 10% $NaHCO_3$ and 10% $NaHSO_4$, and once with brine before being dried with $MgSO_4$, filtered and evaporated. TMP-

G3-[ene]$_{24}$ was obtained as a pale-yellow oil upon concentration (7.9 g, 25 %). ^{1}H-NMR (400 MHz, CD_3OD) δ/ppm: 5.87 (24H, ddt, J = 6, 11, 17 Hz, H18), 5.25 (24H, dq, J = 2, 17 Hz, H19a), 5.14 (24H, dq, J = 2, 11 Hz, H19b), 4.40 – 4.15 (42H, m, H4, H8 and H12), 3.96 (48H, dt, J = 2, 6 Hz, H17), 3.53 (48H, m, H16), 1.61 (2H, t, J = 8 Hz, H2), 1.33 (9H, s, H7), 1.26 (18H, s, H11), 1.20 (36H, s, H15), 1.01 (3H, t, J = 8 Hz, H1). ^{13}C-NMR (101 MHz, CD_3OD) δ/ppm: 175.2 (C5, C9, C13), 173.4 (C5, C9, C13), 173.3 (C5, C9, C13), 136.2 (C18), 117.1 (C19), 73.3 (C17), 66.9 (C4, C8, C12, C16), 66. 1(C4, C8, C12, C16), 65.4 (C4, C8, C12, C16), 49.8 (C6, C10, C14), 48.2 (C6, C10, C14), 48.1 (C6, C10, C14), 42.9 (C3), 18.6 (C7, C11, C15), 18.4 (C7, C11, C15), 8.4 (C1). MALDI-TOF: $[M+Na]^+_{Theo}$: 3554.83 Da, $[M+Na]^+_{Exp}$: 3554.82 Da. $[M+K]^+_{Theo}$: 3570.85 Da, $[M+K]^+_{Exp}$: 3570.82 Da. SEC (DMF) M_n =3208.9 g mol^{-1}, M_w= 3247.9 g mol^{-1}, Đ = 1.01.

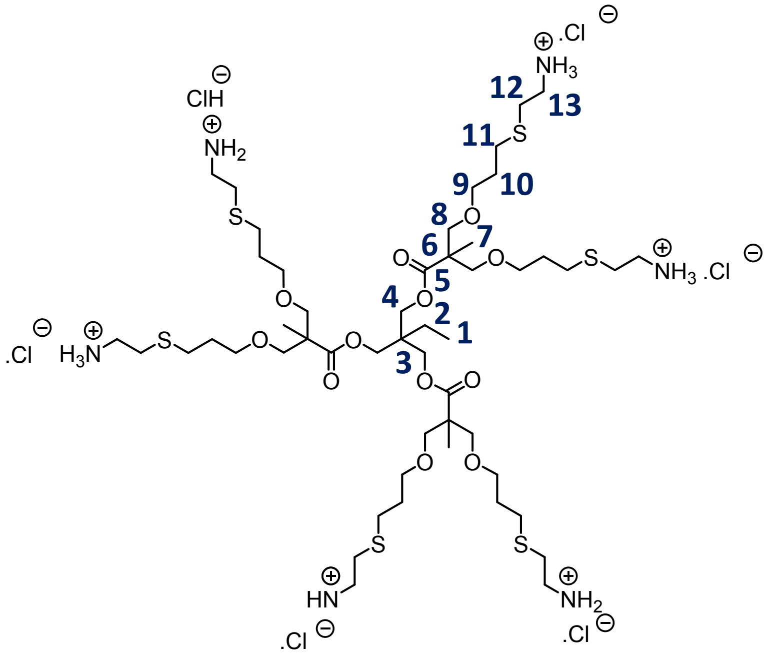

TMP-G1-[Cys]$_6$. In a RBF equipped with a magnetic stirrer, the dendritic precursor TMP-G1-[ene]$_6$ (0.50 g, 0.69 mmol) and the cysteamine hydrochloride (0.54 g, 4.69 mmol) were dissolved in MeOH. The mixture was purged with argon for 10 minutes and then the photoinitiator 2,2-Dimethoxy-2-phenylacetophenone (DMPA) (30 mg) was added in solid state. Afterward, the mixture was irradiated with UV light (365 nm) for 1 hour. Upon completion of the reaction, the crude was concentrated and purified through size exclusion chromatography (Sephadex G-10). The pure fractions were collected and the solvent was completely removed by rotary evaporation followed by freeze-dry. TMP-G1-[Cys]$_6$ was obtained as yellow oil (0.3 g, 31 %).^{1}H-NMR (400 MHz, CD_3OD) δ/ppm: 4.07 (6H, s, H4), 3.54 (24H, m, H8 and H9), 3.15 (12H, t, *J* = 6.9 Hz, H13), 2.83 (12H, m, H12), 2.65 (12H, t, *J* = 7.2 Hz, H11), 1.85 (12H, m, H10), 1.54 (2H, m, H2), 1.21 (9H, s, H7), 0.96 (3H, m, H1). ^{13}C-NMR (101 MHz, CD_3OD) δ/ppm: 175.6 (C5), 74.1 (C8), 70.8 (C9), 64.5 (C4), 50.0 (C6), 43.0 (C3), 40.0 (C13), 30.5 (C12), 29.7 (C11), 29.1 (C10), 24.1 (C2), 18.5 (C7), 8.0 (C1). MALDI-TOF: $[M+H]^+_{Theo}$: 1185.61 Da, $[M+H]^+_{Exp}$: 1185.32 Da. $[M+Na]^+_{Theo}$: 1207.59 Da, $[M+Na]^+_{Exp}$: 1207.25 Da. $[M+K]^+_{Theo}$:1223.7 Da, $[M+K]^+_{Exp}$: 1223.32 Da.

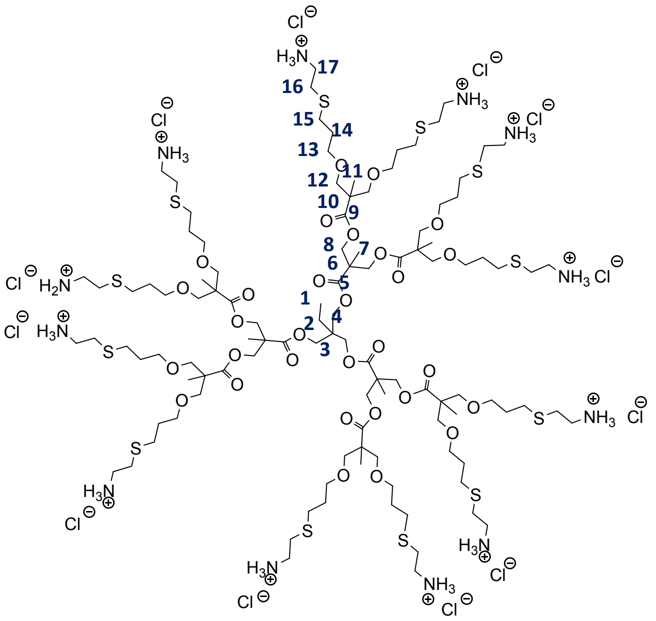

TMP-G2-[Cys]$_{12}$. In a RBF equipped with a magnetic stirrer , the dendritic precursor TMP-G2-[ene]$_{12}$ (1.62 g, 0.976 mmol) and the cysteamine hydrochloride (1.50 g, 13.2 mmol) were dissolved in MeOH. The mixture was purged with argon for 10 minutes and then the photoinitiator DMPA (81 mg) was added in solid state. Afterward, the mixture was irradiated with UV light (365 nm) for 1 hour. Upon completion of the reaction, the crude was concentrated and purified through size exclusion chromatography (Sephadex G-10). The pure fractions were collected and the solvent was completely removed by rotary evaporation followed by freeze-dry. TMP-G2-[Cys]$_{12}$ was obtained as yellow oil (1.5 g, 52 %). ^{1}H-NMR (400 MHz, CD_3OD) δ/ppm: 4.29 (12H, s, H8), 4.16 (6H, s, H4), 3.53 (48H, m, H12 and H13), 3.16 (24H, t, *J = 7.0 Hz*, H17), 2.85 (24H, t, *J* = 7.0 Hz, H16), 2.66 (24H, t, *J = 7.2 Hz*, H15), 1.86 (24H, m, H14), 1.60 (2H, m, H2), 1.30 (9H, s, H7), 1.19 (18H, s, H11), 1.00 (3H, m, H1). ^{13}C-NMR (101 MHz, CD_3OD) δ/ppm: 175.3 (C9), 173.6 (C5), 73.8 (C12), 70.8 (C13), 66.1 (C8), 65.1 (C4), 49.8 (C10), 48.2 (C6), 42.9 (C3), 40.0 (C17), 30.5 (C16), 29.7 (C15), 29.1 (C14), 24.1 (C2), 18.6 (C11), 18.4 (C7), 8.3 (C1). MALDI-TOF: $[M+H]^+_{Theo}$: 2584.27 Da, $[M+H]^+_{Exp}$: 2583.99 Da. $[M+Na]^+_{Theo}$: 2606.25 Da, $[M+Na]^+_{Exp}$: 2605.99 Da, $[M+K]^+_{Theo}$: 2622.36 Da, $[M+K]^+_{Exp}$: 2621.97 Da.

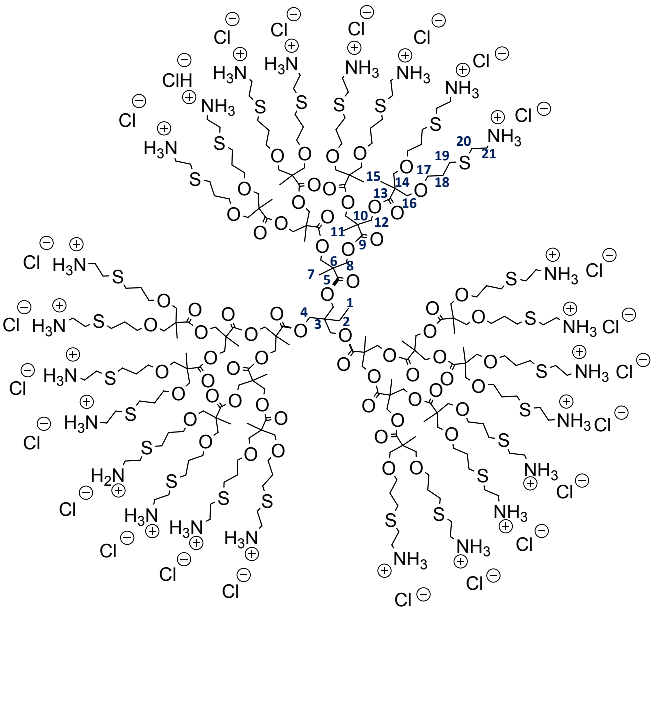

TMP-G3-[Cys]$_{24}$. In a RBF equipped with a magnetic stirrer, the dendritic precursor TMP-G3-ene]$_{24}$ (1.00 g, 0.28 mmol) and the cysteamine hydrochloride (0.87 g, 7.67 mmol) were dissolved in MeOH. The mixture was purged with argon for 10 minutes and then the photoinitiator DMPA (240 mg) was added in solid state. Afterward, the mixture was irradiated with UV light (365 nm) for 1 hour. Upon completion of the reaction, the crude was concentrated and purified through size exclusion chromatography (Sephadex G-10). The pure fractions were collected and the solvent was completely removed by rotary evaporation followed by freeze-dry. TMP-G3-[Cys]$_{24}$ was obtained as yellow oil (0.6 g, 35 %). ^{1}H-NMR (400 MHz, CD_3OD) δ/ppm: 4.42 – 4.16 (42H, m, H4, H8 and H12), 3.56 (96H, m, H16 and H17), 3.19 (48H, m, H21), 2.88 (48H, m, H20), 2.69 (48H, m, H19), 1.89 (48H, m, H18), 1.65 (2H, m, H2), 1.39 (9H, s, H7), 1.29 (18H, s, H11), 1.21 (36H, s, H15), 1.04 (3H, m, H1). ^{13}C-NMR (101 MHz, CD_3OD) δ/ppm: 175.3 (C13), 173.5 (C9), 173.3 (C5), 73.7 (C16), 70.8 (C17), 66.0 (C4, C8 and C12), 49.7-48.1 (C6 and C10), 40.1 (C21), 30.6 (C20), 29.7 (C19), 29.1 (C18), 24.1 (C2), 18.7-18.5 (C7, C11 and C15). MALDI-TOF: $[M+H]^{+}_{Theo}$: 5381.56 Da, $[M+H]^{+}_{Exp}$: 5385.9 Da.

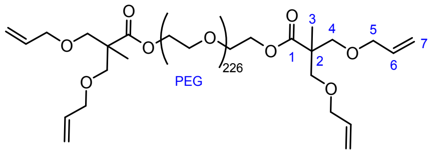

PEG10k-G1-[ene]$_4$. PEG10K (10 g, 0.001 mol) was dissolved in DCM (15 mL) and pyridine (0.5 mL, 0.06 mol) with stirring. DMAP (73 mg, 0.6 mmol) was added, followed by BAPA acetonide anhydride (13) (2 g, 0.005 mol) under vigorous stirring at room temperature. The reaction was allowed to proceed for 16 h and monitored by ^{13}C-NMR and ^{1}H- NMR spectroscopy. Upon completion the crude product was precipitated from the reaction mixture by the addition of ether three times. The product was isolated by filtration and dried in vacuo to give PEG10k-G1-[ene]$_4$ as a white powder (8 g, 77%). ^{1}H-NMR ($CDCl_3$, 400 MHz) δ/ppm: 5.85 (4H, ddt, J=5, 11, 17 Hz, H6), 5.19 (8H, m, H7), 4.24 (4H, m, H4, 3.96 (8H, m, H5), 3.82-3.44 (914 H, m, H4, PEG), 1.21 (6H, s, H3). ^{13}C-NMR ($CDCl_3$, 101 MHz), δ/ppm: 174.6 (C1), 134.9 (C6), 116.5 (C7), 72.3 (C4), 72.1 (C5), 70.6 (PEG), 69.2 (PEG), 63.7 (PEG), 48.4 (C2), 18.0 (C3). SEC (DMF): M_n =12483 g mol^{-1}, M_w= 12687 g mol^{-1}, Đ = 1.01.

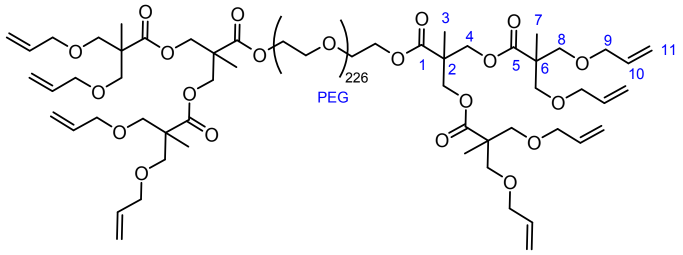

PEG10k-G2-[ene]$_8$. PEG10k-G1-[OH]$_4$ (15 g, 1.5 mmol) was dissolved in DCM (50 mL) and pyridine (1.3 mL, 0.015 mol) with stirring. DMAP (0.2 g, 1.5 mmol) was added, followed by BAPA acetonide anhydride (5 g, 12.5 mmol) under vigorous stirring at room temperature. The reaction was allowed to proceed for 16 h and monitored by ^{13}C-NMR and ^{1}H- NMR spectroscopy. Upon completion the crude product was precipitated from the reaction mixture by the addition of ether three times. The product was isolated by filtration and dried *in vacuo* to give PEG10k-G2-[ene]$_8$ as a white powder (14.4 g, 87%). ^{1}H-NMR ($CDCl_3$, 400 MHz) δ/ppm: 5.81 (8H, ddt, *J*=5, 11, 17 Hz, H10), 5.16 (16H, m, H11), 4.34-4.15 (16H, m, H4, H8a), 3.97-3.88 (16H, m, H9), 3.80-3.42 (922 H, m, H8b, PEG), 1.21 (6H, s, H3), 1.16 (12H, s, H7). ^{13}C-NMR ($CDCl_3$, 101 MHz), δ/ppm: 173.9 (C1), 172.7 (C5), 134.8 (C10), 116.7 (C11), 72.3 (C8), 72.0 (C9), 70.6 (PEG), 65.3 (C4), 64.1 (PEG), 48.5 (C6), 46.7 (C2), 18.0 (C7), 17.6 (C3). SEC (DMF): M_n = 12861 g mol^{-1}, M_w= 13168 g mol^{-1}, Đ =1.02.

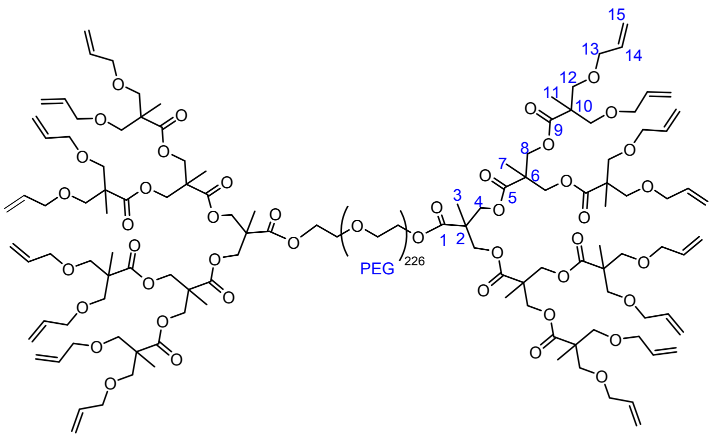

PEG10k-G3-[ene]$_{16}$. PEG10k-G2-[OH]$_8$ (5 g, 0.5 mmol) was dissolved in DCM (10 mL) and pyridine (9.9 mL, 1.1 mmol) with stirring. DMAP (135 mg, 1.1 mmol) was added, followed by BAPA acetonide anhydride (**13**) (4.4 g, 11 mmol) under vigorous stirring at room temperature. The reaction was allowed to proceed for 16 h and monitored by ^{13}C-NMR and ^{1}H- NMR spectroscopy. Upon completion the crude product was precipitated from the reaction mixture by the addition of ether three times. The product was isolated by filtration and dried *in vacuo* to give PEG10k-G3-[ene]$_{16}$ as a white powder (4.2 g, 73%). ^{1}H-NMR ($CDCl_3$, 400 MHz) δ/ppm: 5.78 (16H, ddt, *J*=5, 11, 17 Hz, H14), 5.13 (32H, m, H15), 4.28-4.10 (28H, m, H4, H8, H12), 3.97-3.83 (32H, m, H13), 3.77-3.39 (938 H, m, H4, H8, H12, PEG), 1.24-1.04 (18H, m, H3, H7, H11). ^{13}C-NMR ($CDCl_3$, 101 MHz), δ/ppm: 173.7 (C1, C5, C9), 171.9 (C1, C5, C9), 134.7 (C14), 116.6 (C15), 72.2 (C12), 71.9 (C13), 70.5 (PEG), 65.8 (C8), 64.8 (C4), 64.3 (PEG), 48.4 (C10), 46.7 (C6), 46.7 (C2), 17.9 (C11), 17.5 (C7), 15.2 (C3). SEC (DMF): M_n = 14357 g mol^{-1}, M_w= 16026 g mol^{-1}, Đ =1.10.

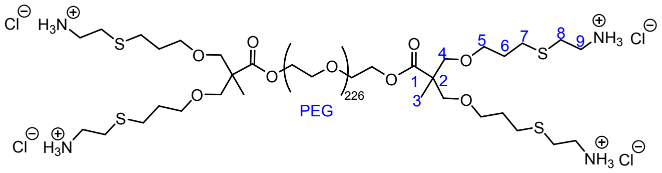

PEG10k-G1-[Cys]$_6$. The DLD precursor PEG10k-G1-[ene]$_6$ (1.50 g, 0.14 mmol) and the cysteamine hydrochloride (0.13 g, 1.15 mmol) were dissolved in MeOH. The mixture was purged with argon for 10 minutes and then the photoinitiator DMPA (100 mg) was added in solid state. Afterward, the mixture was irradiated with UV light (365 nm) for 1 hour. Upon completion of the reaction, the crude was concentrated and purified through size exclusion chromatography (Sephadex G-10). The pure fractions were collected and the solvent was completely removed by rotary evaporation followed by freeze-dry. PEG10k-G1-[Cys]$_6$ was obtained as yellow oil (1.10 g, 70 %). ^{1}H-NMR (400 MHz, CD_3OD) δ/ppm: 4.29-4.19 (4H, m, H4), 3.86-3.40 (918H, m, PEG and H5), 3.22 (8H, t, H9), 2.83 (8H, t, H8), 2.66 (8H, t, H7), 1.85 (8H, m, H6), 1.22 (6H, s, H3). ^{13}C-NMR ($CDCl_3$, 101 MHz), δ/ppm: 175.7 (C1), 74.2 (C4), 73.6 (C5), 71.4-64.6 (PEG), 50.3 (C2), 40.3 (C9), 30.5 (C8), 29.6 (C7), 29.1 (C6), 18.3(C3). SEC (DMF): M_n =11299 g mol^{-1}, M_w= 13111 g mol^{-1}, Đ = 1.16.

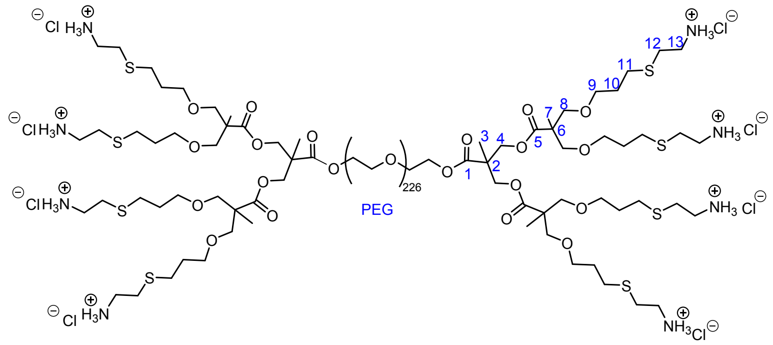

PEG10k-G2-[Cys]₁₂. The DLD precursor PEG10k-G2-[ene]$_{12}$ (1.00 g, 0.091 mmol) and the cysteamine hydrochloride (0.16 g, 1.45 mmol) were dissolved in MeOH. The mixture was purged with argon for 10 minutes and then the photoinitiator DMPA (50 mg) was added in solid state. Afterward, the mixture was irradiated with UV light (365 nm) for 1 hour. Upon completion of the reaction, the crude was concentrated and purified through size exclusion chromatography (Sephadex G-10). The pure fractions were collected and the solvent was completely removed by rotary evaporation followed by freeze-dry. PEG10k-G2-[Cys]$_{12}$ was obtained as yellow oil (0.5 g, 46 %). ^{1}H-NMR (400 MHz, CD_3OD) δ/ppm: 4.40-4.22 (24H, m, H4, H8, H9), 3.89-3.44 (926H, m, PEG, H4, H8, H9), 3.22 (16H, t, H13), 2.84 (16H, t, H12), 2.66 (16H, t, H11), 1.85 (16H, m, H10), 1.30 (6H, s, H3), 1.20 (12H, s, H7). ^{13}C-NMR ($CDCl_3$, 101 MHz), δ/ppm: 175.1-174.0 (C1, C5), 75.1-73.4 (C4, C8, C9), 71.5-65.5 (PEG), 50.0 (C2, C6), 40.3 (C13), 30.5 (C12), 29.7 (C11), 29.2 (C10), 18.8-18.2 (C3, C7). SEC (DMF): M_n =11817 g mol^{-1}, M_w= 15485 g mol^{-1}, Đ = 1.31.

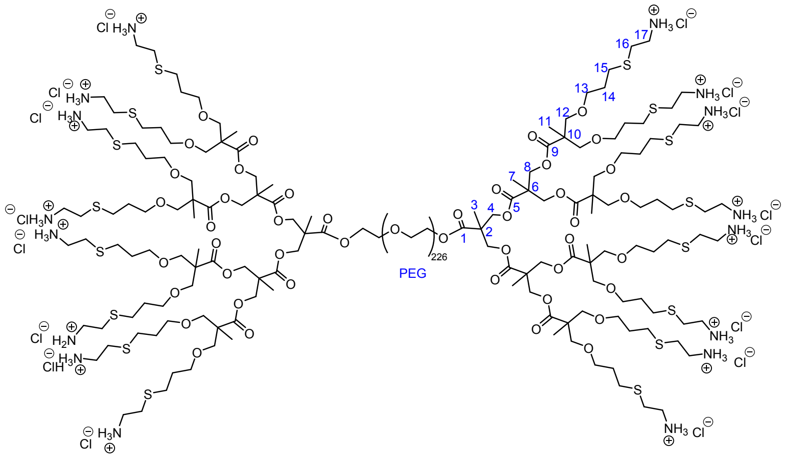

PEG10k-G3-[Cys]₂₄. The DLD precursor PEG10k-G3-[ene]$_{24}$ (1.14 g, 0.093 mmol) and the cysteamine hydrochloride (0.34 g, 2.97 mmol) were dissolved in MeOH. The mixture was purged with argon for 10 minutes and then the photoinitiator DMPA (60 mg) was added in solid state. Afterward, the mixture was irradiated with UV light (365 nm) for 1 hour. Upon completion of the reaction, the crude was concentrated and purified through size exclusion chromatography (Sephadex G-10). The pure fractions were collected and the solvent was completely removed by rotary evaporation followed by freeze-dry. PEG10k-G3-[Cys]$_{24}$ was obtained as yellow oil (0.6 g, 43 %). ^{1}H-NMR (400 MHz, CD_3OD) δ/ppm: 4.38-4.17 (32H, m, H4, H8, H12, H13), 3.86-3.40 (934H, m, PEG, H4, H8, H12, H13), 3.21 (32H, t, H17), 2.85 (32H, t, H16), 2.67 (32H, t, H15), 1.86 (32H, m, H14), 1.35 (6H, s, H3), 1.29 (12H, s, H7), 1.20 (24H, s, H11). ^{13}C-NMR ($CDCl_3$, 101 MHz), δ/ppm: 175.0-173.4 (C1, C5, C9), 74.6-73.2 (C4, C8, C12, C13), 71.4-64.5 (PEG), 50.3-49.7 (C2, C6, C10), 40.3 (C17), 30.5 (C16), 29.5 (C15), 29.1 (C14), 18.8-18.1 (C3, C7, C11). SEC (DMF): M_n =12250 g mol^{-1}, M_w= 15179g mol^{-1}, Đ = 1.24.

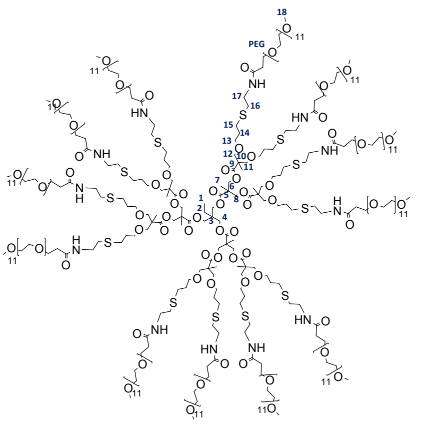

TMP-G2-[Cys-mPEG$_{11}$]$_{12}$. TMP-G2-[Cys]$_{12}$ (20 mg, 0.077 mmol) was deprotonated with $NaHCO_3$ to pH 8 and after dissolved in pyridine (3 mL) with stirring. DMAP (3. 9 mg, 0.32 mmol) was added followed by mPEG anhydride (158 mg, 0.134 mmol) dissolved in DCM (5mL) and at room temperature. The reaction proceeded for 16 h and monitored by ^{1}H-NMR and MALDI. Upon completion of the reaction, the crude was concentrated, dissolved in ethanol (96 %) and purified through dialysis (1000 exclusion limit). The pure compound was concentrated by rotary evaporation followed by freeze-dry. TMP-G2-[Cys-mPEG$_{11}$]$_{12}$ was obtained as a sticky yellow solid (32 mg, 44 %). ^{1}H-NMR (400 MHz, CD_3OD) δ/ppm: 4.32 (12H, s, H8), 4.19 (6H, s, H4), 3.66 (624H, s, PEG, H12, H13), 3.38 (36H, s, H18), 2.65 (48H, m, H15 and H16), 2.49 (24H, m, , H17), 1.84 (24H, m, H14), 1.32 (9H, s, H7), 1.21 (18H, s, H11), 1.03 (3H, m, H1).). MALDI-TOF: $[M+K]^+_{Theo}$: 9466.16 Da, $[M+K]^+_{Exp}$: 9503.53 Da.

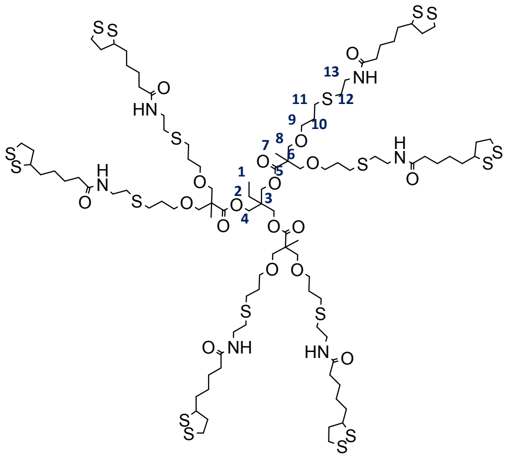

TMP-G1-[Cys-lipoic acid]$_6$. CDI (24.5 mg, 0.151 mmol) was slowly added over a solution of α-lipoic acid (31.2 mg, 0.151 mmol) in DCM. After 1 hour of stirring at room temperature, the crude mixture was added to a solution of the TMP-G1-[Cys]$_6$ (20 mg, 0.017 mmol) previously deprotonated with $NaHCO_3$ to pH 8. The mixture was kept under stirring overnight. The reaction was monitored by ^{1}H-NMR and MALDI. Upon completion of the reaction, the crude was concentrated, dissolved in ethanol (96 %) and purified through dialysis (100- 500 exclusion limit). The pure compound was concentrated by rotary evaporation followed by freeze-dry. TMP-G2-[Cys-lipoic acid]$_{12}$ was obtained as a yellow solid (23 mg, 59 %). MALDI-TOF: $[M+H]^+_{Theo}$: 2312.80 Da, $[M+H]^+_{Exp}$: 2313.41 Da. $[M+Na]^+_{Theo}$: 2335.79 Da, $[M+Na]^+_{Exp}$: 2335.53 Da. $[M+K]^+_{Theo}$:2351.90 Da, $[M+K]^+_{Exp}$: 2351.56 Da.

Figures

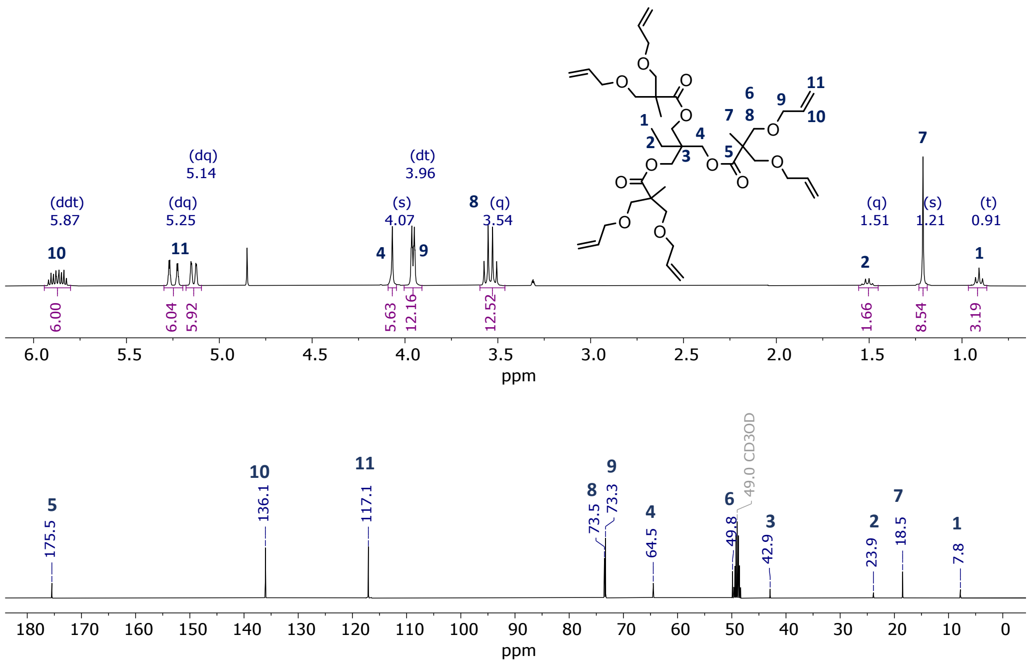

Figure S1. ^{1}H and ^{13}C NMR spectra of the dendrimer TMP-G1-[ene]$_6$ in CD_3OD.

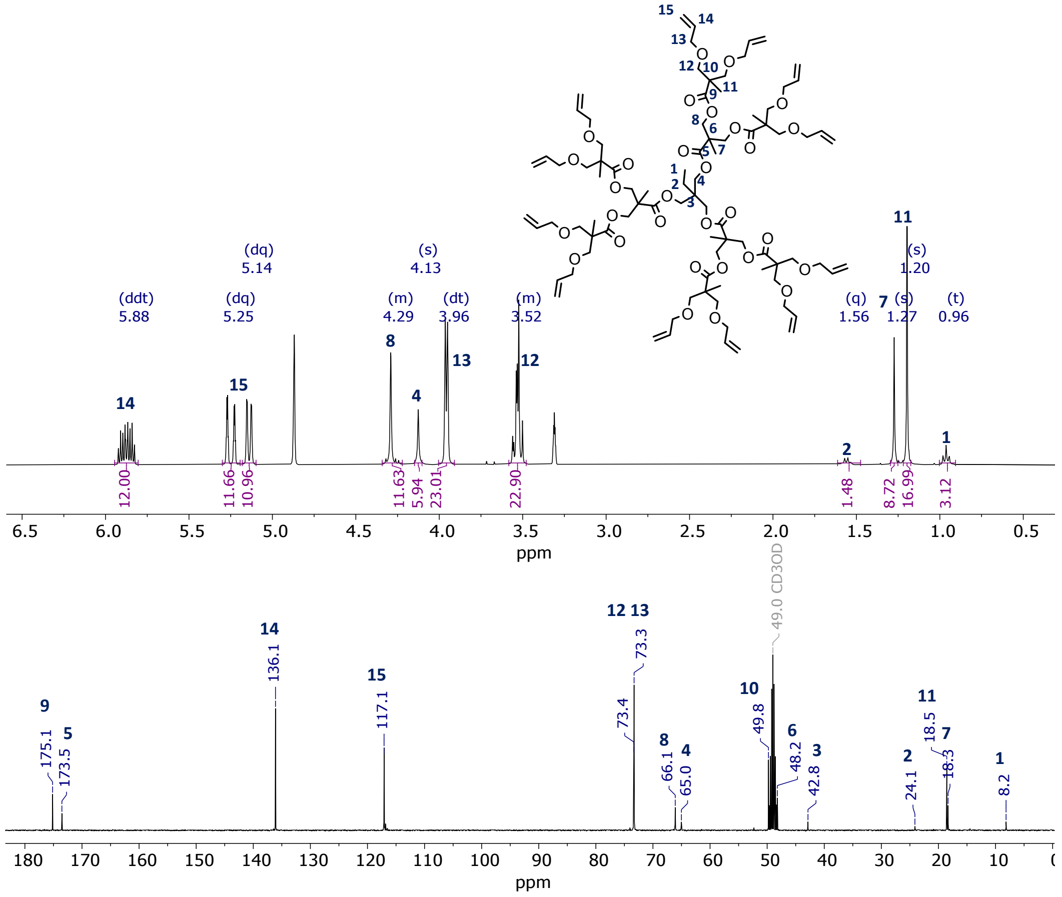

Figure S2. ^{1}H and ^{13}C NMR spectra of the dendrimer TMP-G2-[ene]$_{12}$ in CD_3OD.

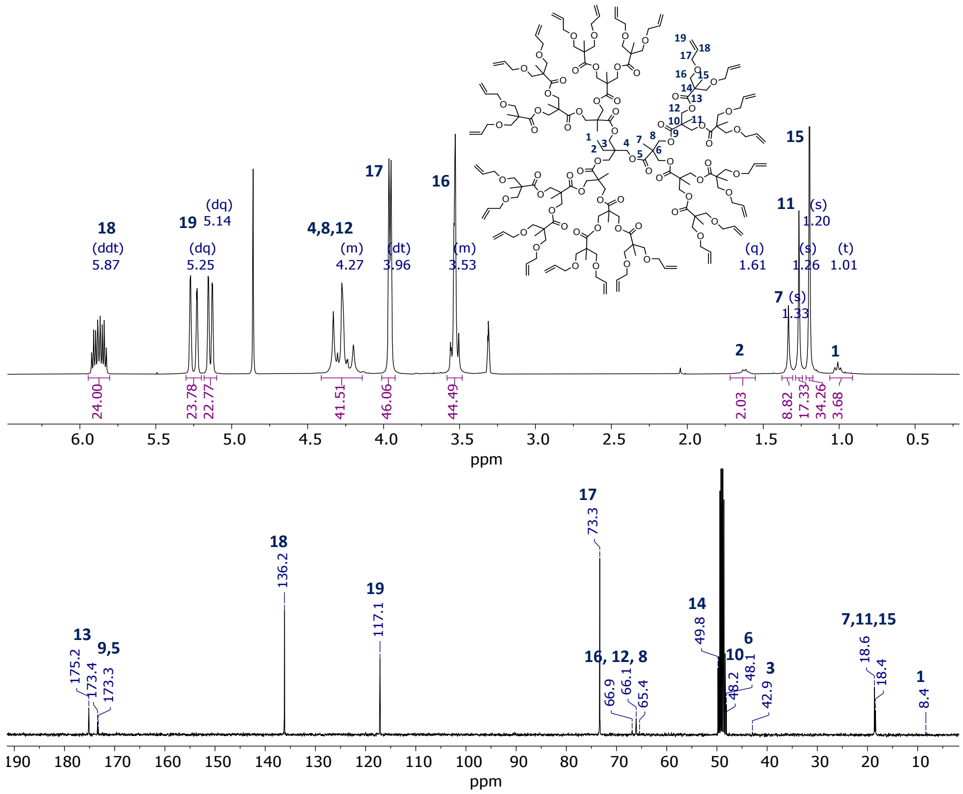

Figure S3. ^{1}H and ^{13}C NMR spectra of the dendrimer TMP-G3-[ene]$_{24}$ in CD_3OD.

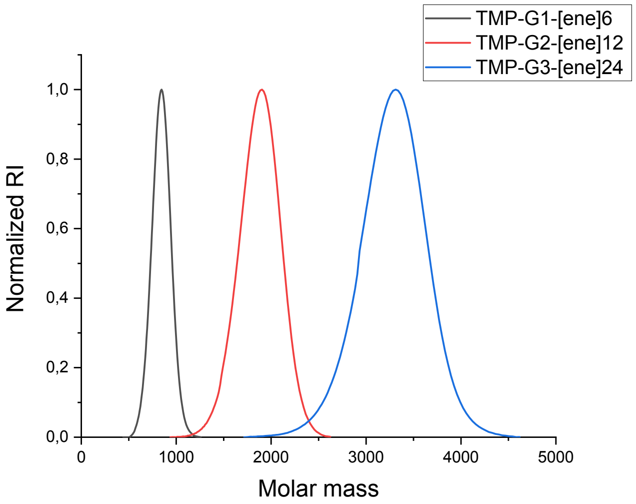

Figure S4. SEC overlay of TMP-G1-[ene]$_6$, TMP-G2-[ene]$_{12}$ and TMP-G3-[ene]$_{24}$

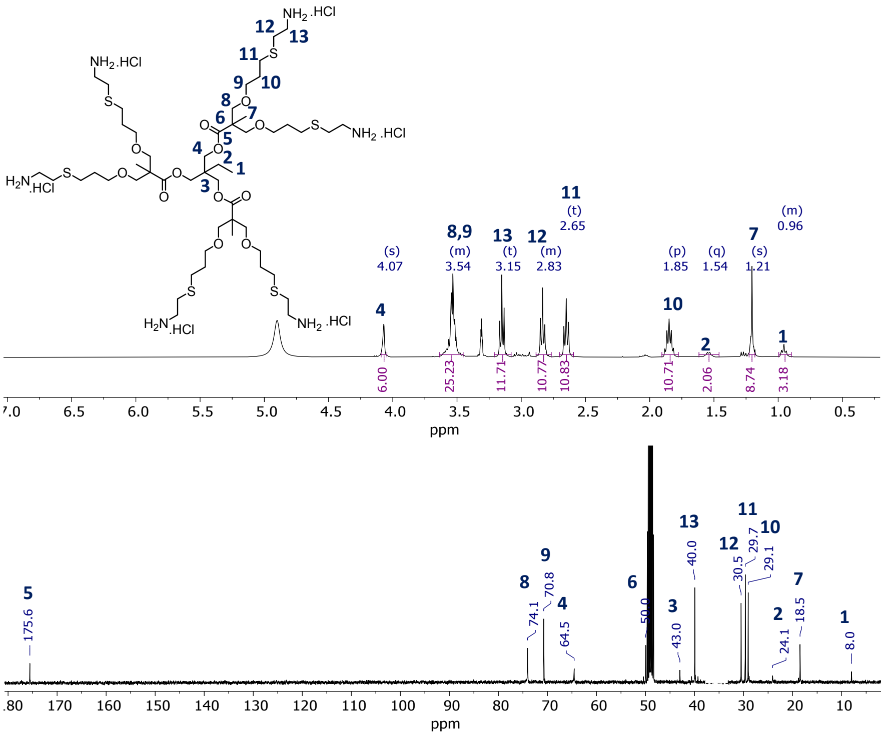

Figure S5. ^{1}H and ^{13}C NMR spectra of the dendrimer TMP-G1-[Cys]$_6$ in CD_3OD.

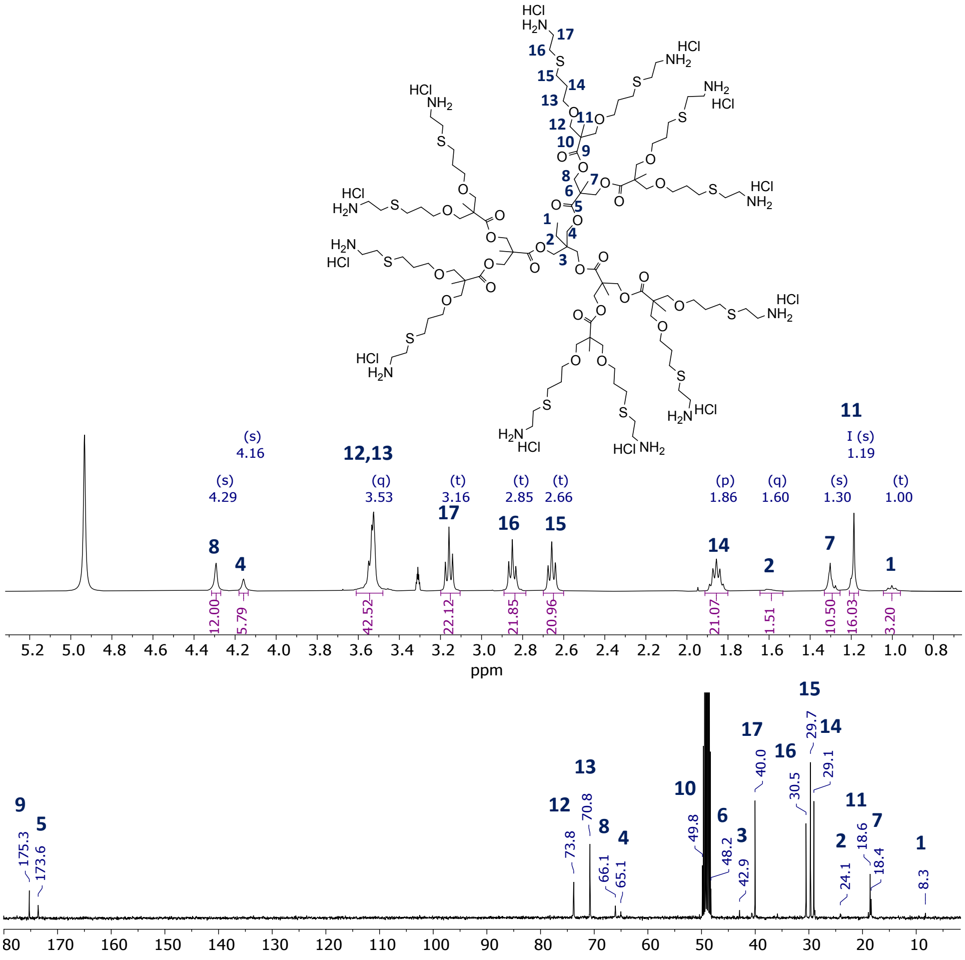

Figure S6. ^{1}H and ^{13}C NMR spectra of the dendrimer TMP-G2-[Cys]$_{12}$ in CD_3OD.

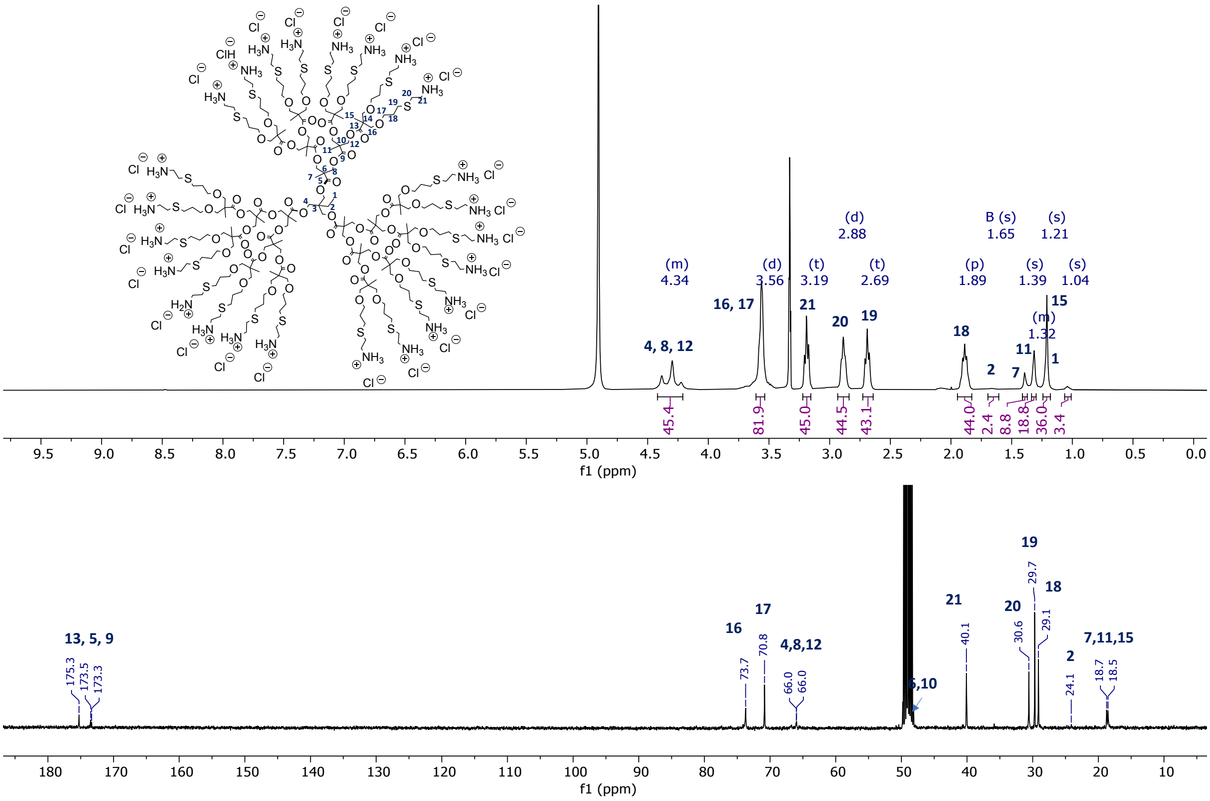

Figure S7. ^{1}H and ^{13}C NMR spectra of the dendrimer TMP-G3-[Cys]$_{24}$ in CD_3OD.

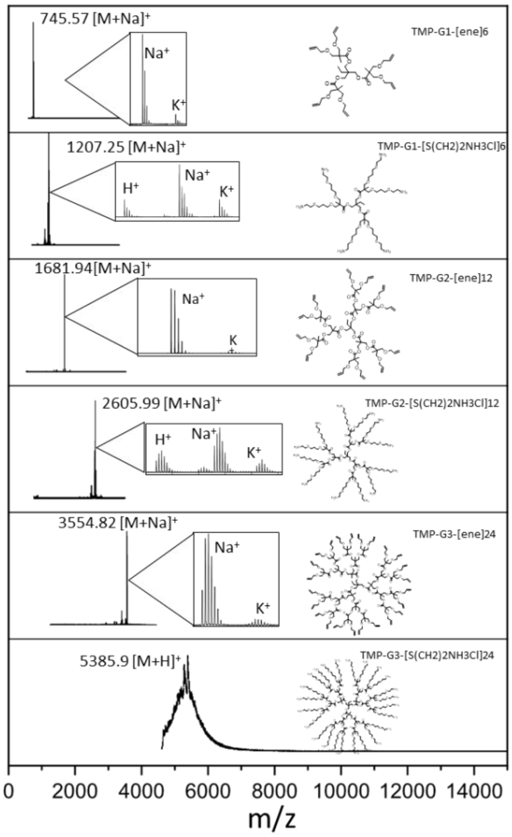

Figure S8. MALDI-ToF spectra of the dendrimers TMP-G1-[ene]$_6$, TMP-G2-[ene]$_{12}$, TMP-G3-[ene]$_{24}$, TMP-G1-[Cys]$_6$, TMP-G2-[Cys]$_{12}$ and TMP-G3-[Cys]$_{24}$.

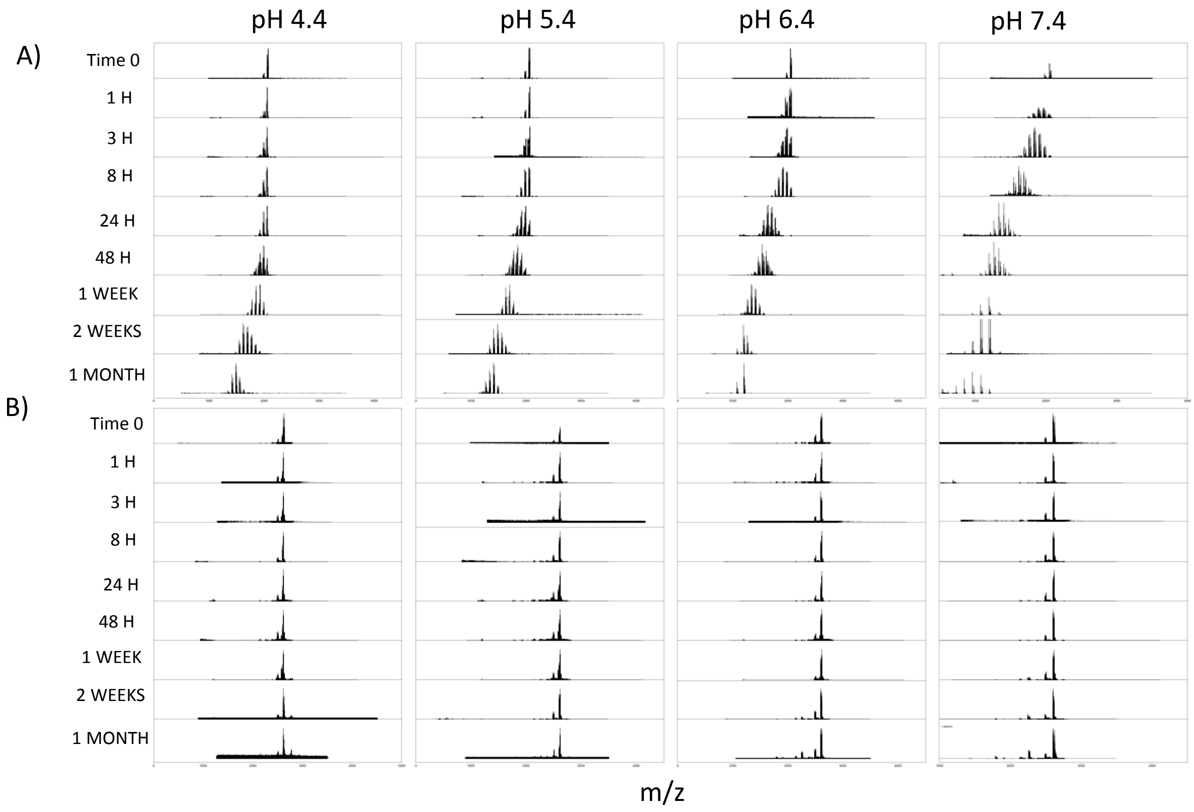

Figure S9. Degradation evaluation through MALDI-ToF of the dendrimers a) TMP-G2-[β-alanine]$_{12}$ and b) TMP-G2-[Cys]$_{12}$ at different pH and times.

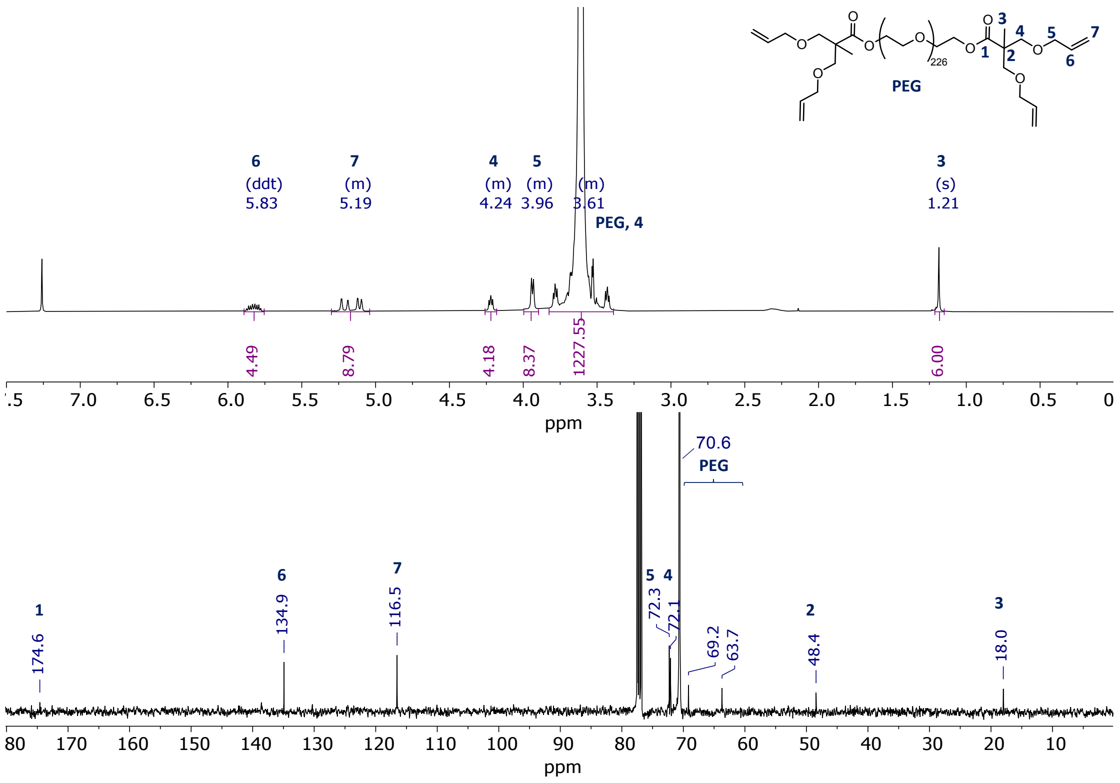

Figure S10. ^{1}H and ^{13}C NMR spectra of the PEG10K-G1-[ene]$_4$ in $CDCl_3$.

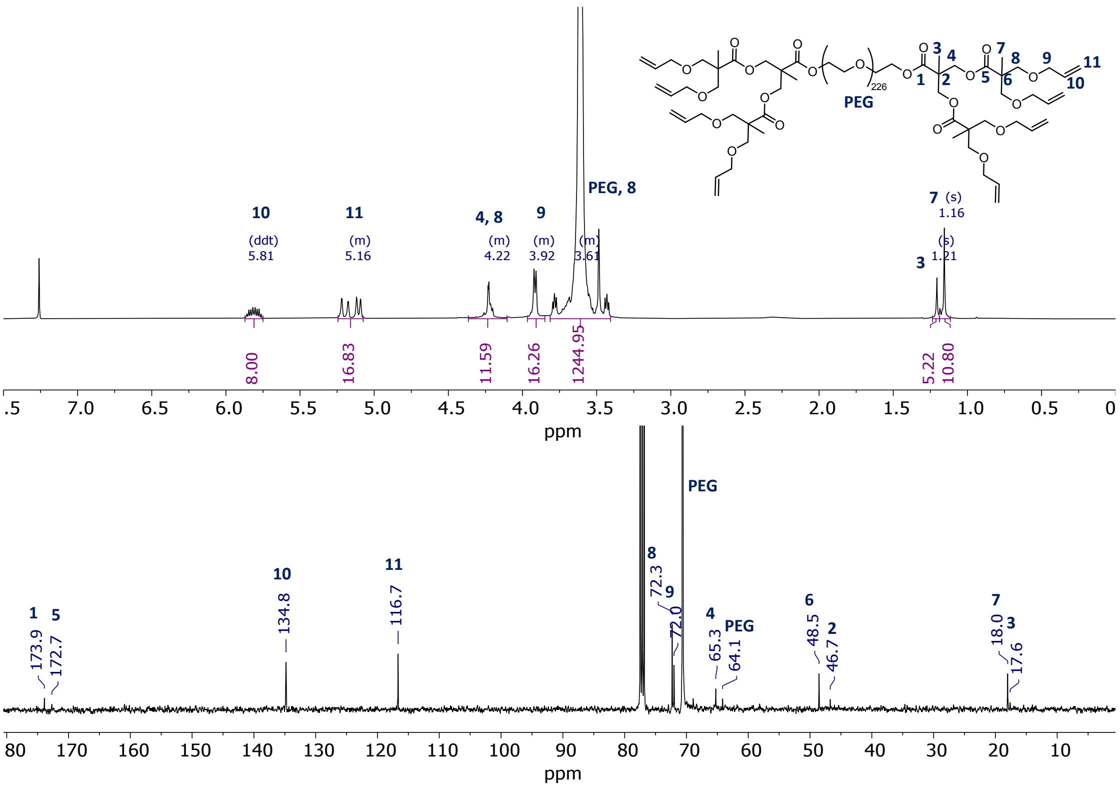

Figure S11. ^{1}H and ^{13}C NMR spectra of the PEG10K-G2-[ene]$_8$ in $CDCl_3$.

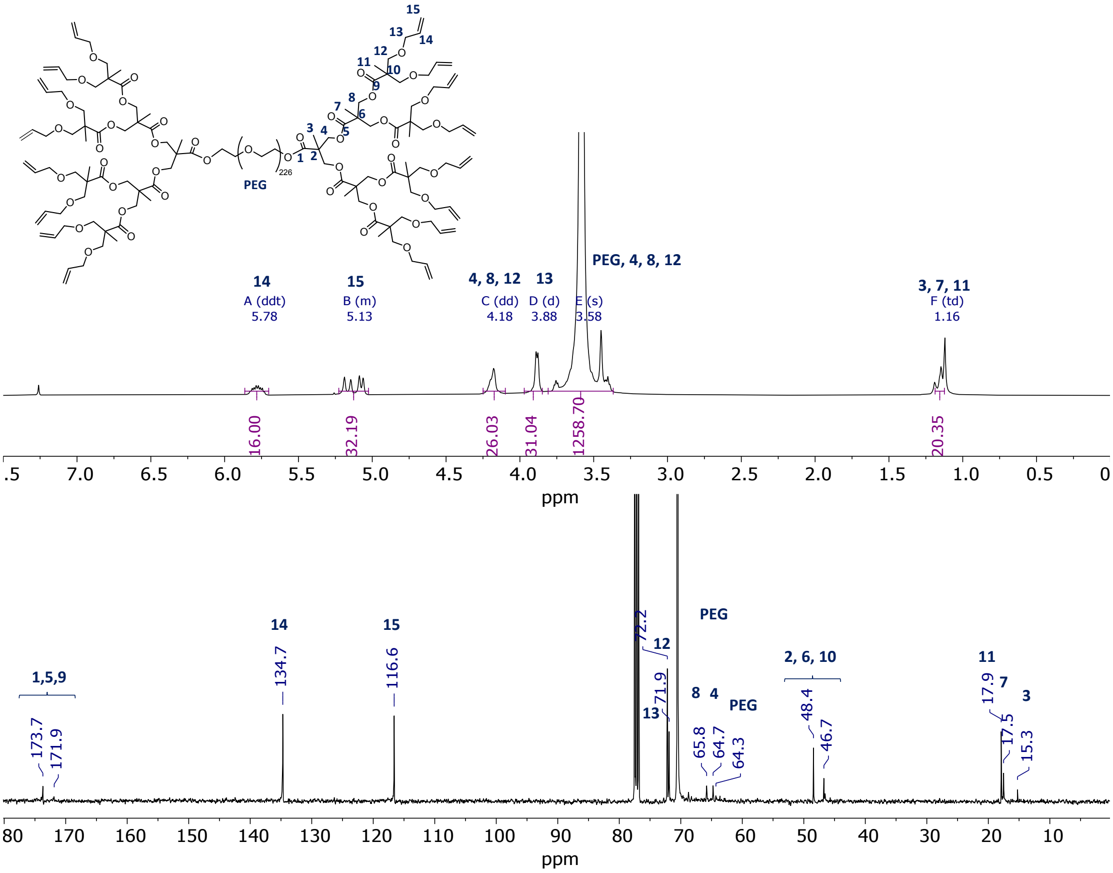

Figure S12. ^{1}H and ^{13}C NMR spectra of the PEG10K-G3-[ene]$_{16}$ in $CDCl_3$.

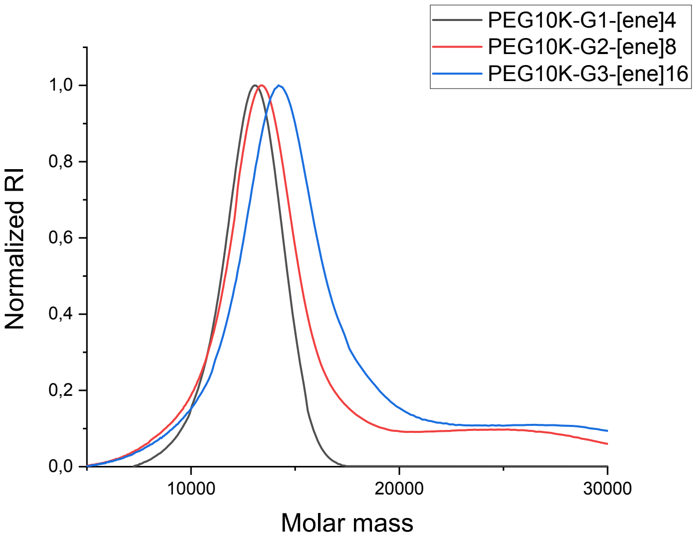

Figure S13. SEC overlay of PEG10K-G1-[ene]$_4$, PEG10K-G2-[ene]$_8$ and PEG10K-G3-[ene]$_{16}$.

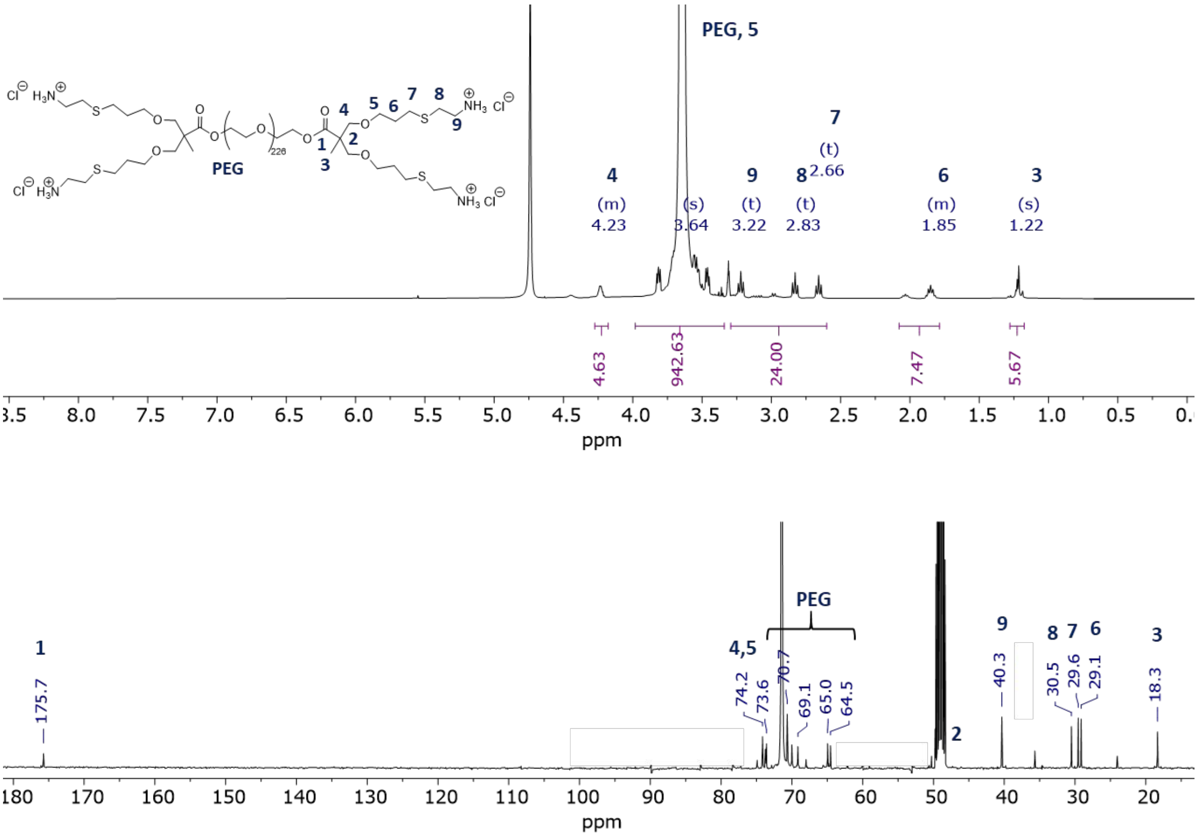

Figure S14. ^{1}H and ^{13}C NMR spectra of the PEG10K-G1-[Cys]$_4$ in CD_3OD.

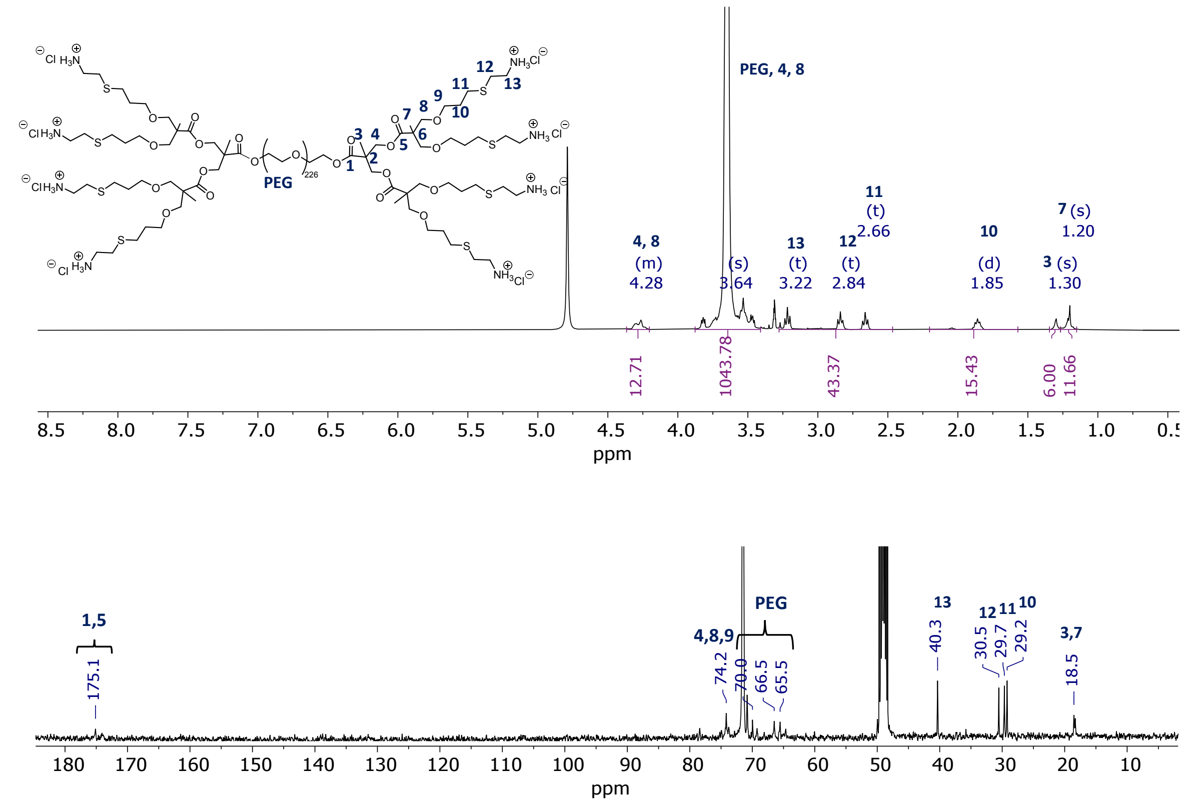

Figure S15. ^{1}H and ^{13}C NMR spectra of the PEG10K-G2-[Cys]$_8$ in CD_3OD.

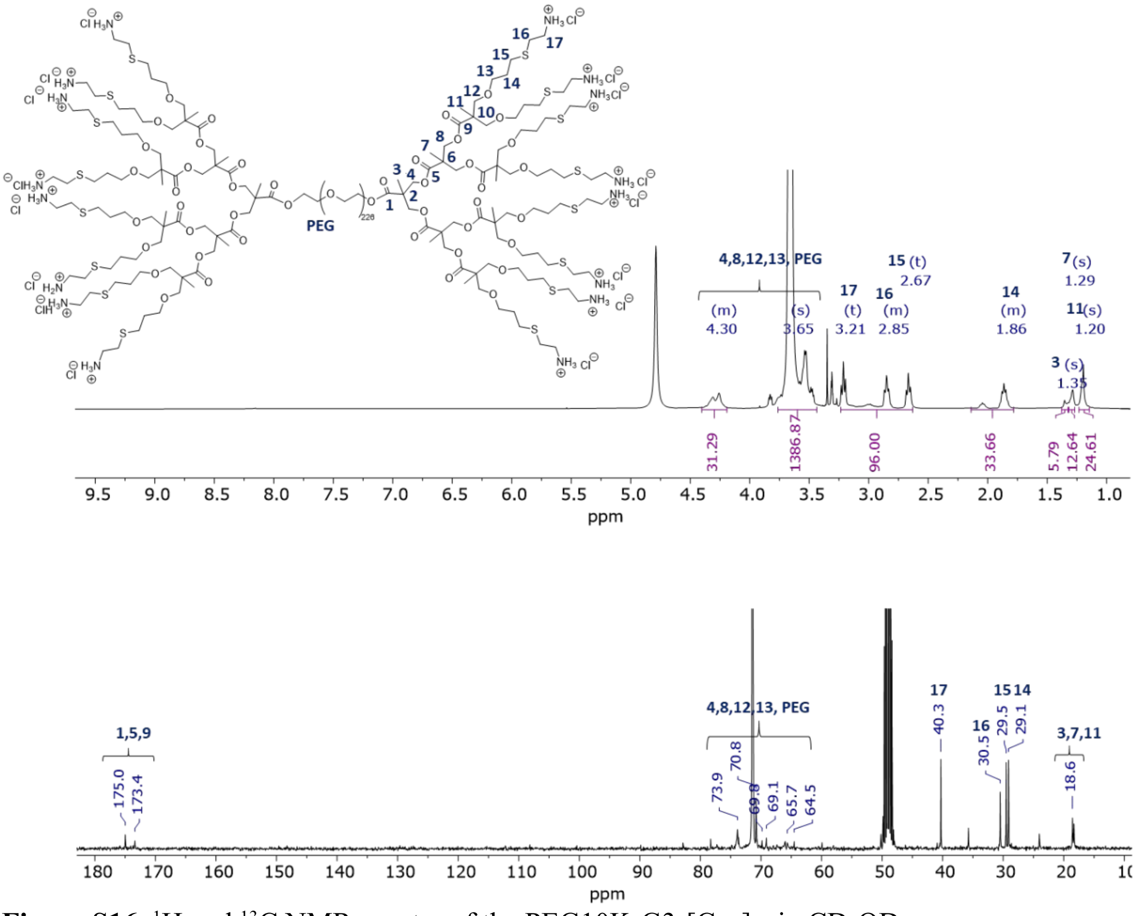

Figure S16. ^{1}H and ^{13}C NMR spectra of the PEG10K-G3-[Cys]$_{16}$ in CD_3OD.

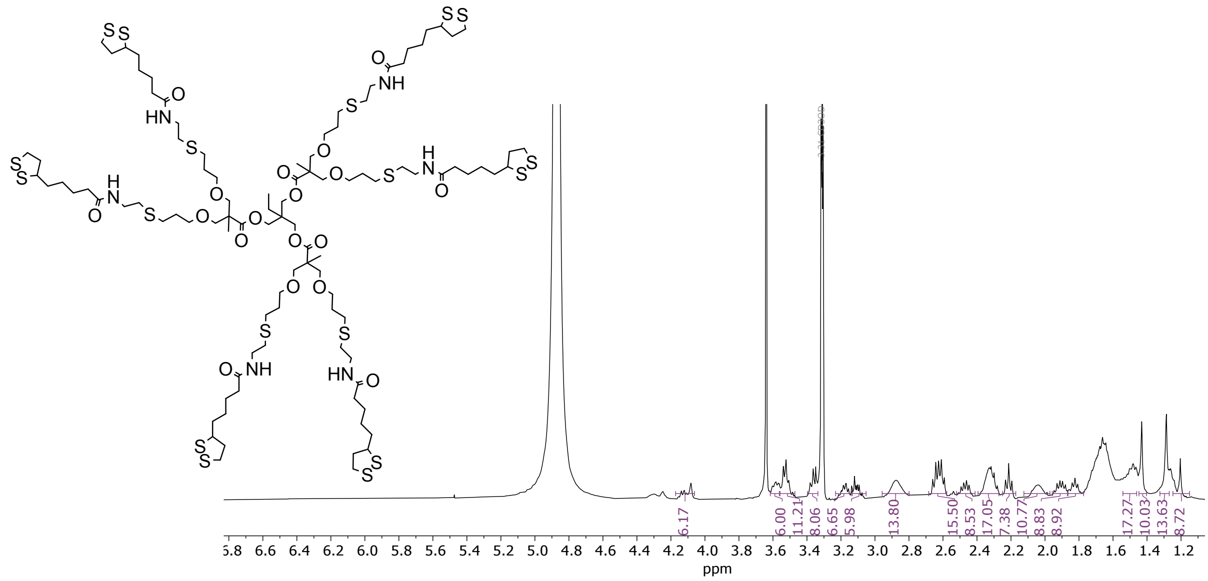

Figure S17. ^{1}H NMR spectrum of the TMP-G1-[Cys-lipoic acid]$_6$.

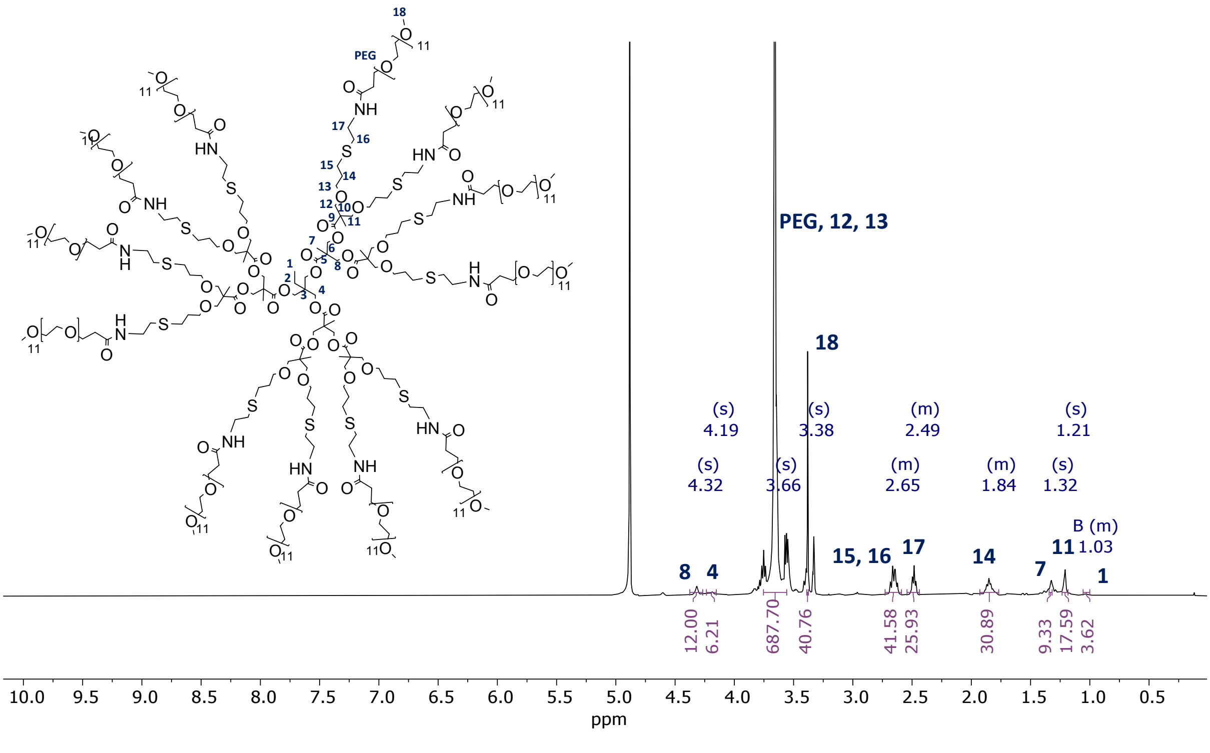

Figure S18. ^{1}H NMR spectrum of the TMP-G2-[Cys-mPEG$_{11}$]$_{12}$.

References

1. Montañez, M. I.; Campos, L. M.; Antoni, P.; Hed, Y.; Walter, M. V.; Krull, B. T.; Khan, A.; Hult, A.; Hawker, C. J.; Malkoch, M., Accelerated Growth of Dendrimers via Thiol−Ene and Esterification Reactions. *Macromolecules* **2010,** *43* (14), 6004-6013.

2. García-Gallego, S.; Hult, D.; Olsson, J. V.; Malkoch, M., Fluoride-Promoted Esterification with Imidazolide-Activated Compounds: A Modular and Sustainable Approach to Dendrimers. *Angew. Chem. Int. Ed.* **2015,** *54* (8), 2416-2419.

3. Andrén, O. C.; Ingverud, T.; Hult, D.; Håkansson, J.; Bogestål, Y.; Caous, J. S.; Blom, K.; Zhang, Y.; Andersson, T.; Pedersen, E., Antibiotic-Free Cationic Dendritic Hydrogels as Surgical-Site-Infection-Inhibiting Coatings. *Adv. Healthcare Mater.* **2019,** *8* (5), 1801619.

4. Zhang, Y.; Mesa-Antunez, P.; Fortuin, L.; Andrén, O. C.; Malkoch, M., Degradable High Molecular Weight Monodisperse Dendritic Poly (ethylene glycols). *Biomacromolecules* **2020,** *21* (10), 4294-4301.
